# Connectivity-Dependent Conductance of 2,2′-Bipyridine-Based Metal Complexes

**DOI:** 10.1021/acsomega.3c06555

**Published:** 2023-12-14

**Authors:** Yahia Chelli, Nicolò Ferri, Andrea Vezzoli, Ross J. Davidson, James Morris, Richard J. Nichols, Simon J. Higgins, Sara Sangtarash, Hatef Sadeghi, Dmitry S. Yufit, Andrew Beeby

**Affiliations:** † Department of Chemistry, Durham University, South Road, Durham DH1 3LE, U.K.; ‡ School of Engineering, University of Warwick, Library Road, Coventry CV4 7AL, U.K.; § Department of Chemistry, University of Liverpool, Crown Street, Liverpool L69 7ZD, U.K.

## Abstract

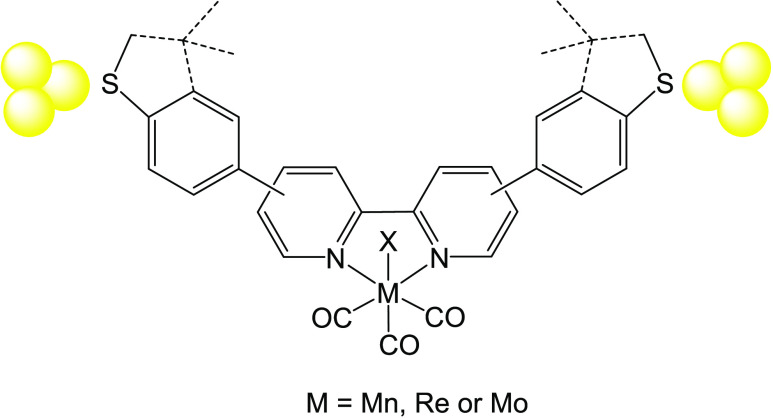

The present work provides an insight into the effect of connectivity isomerization of metal-2,2′-bipyridine complexes. For that purpose, two new 2,2′-bipyridine (bpy) ligand systems, 4,4′-bis(4-(methylthio)phenyl)-2,2′-bipyridine (L^meta^) and 5,5′-bis(3,3-dimethyl-2,3-dihydrobenzothiophen-5-yl)-2,2′-bipyridine (L^para^) were synthesized and coordinated to rhenium and manganese to obtain the corresponding complexes MnL^meta^(CO)_3_Br, ReL^meta^(CO)_3_Br, MnL^para^(CO)_3_Br, MoL^para^(CO)_4_ and ReL^para^(CO)_3_Br. The experimental and theoretical results revealed that coordination to the para system, i.e., the metal ion peripheral to the conductance path, gave a slightly increased conductance compared to the free ligand attributed to the reduced highest occupied molecular orbital (HOMO)–least unoccupied molecular orbital (LUMO) gap. The meta-based system formed a destructive quantum interference feature that reduced the conductance of a S···S contacted junction to below 10^–5.5^*G*_o_, reinforcing the importance of contact group connectivity for molecular wire conductance.

## Introduction

The ability to study conductance at the molecular level has allowed structural and functional relations to be examined. The conductance of single molecules is reliant on the formation of electrode|molecule|electrode junctions; to facilitate this, the molecules need to contain anchor groups capable of electronically coupling the molecule to the electrode, e.g., pyridine or thiol. The importance of the anchor type has been well established.^1^ The dependence of the linkage isomerization has recently been demonstrated; for simple systems such as bis(pyridin-4-ylethynyl)benzene, the *meta*-isomer has a significantly lower conductance than the *para*-isomer.^2^ This behavior can be explained by the quantum interference occurring due to the differing conductance path lengths of the central benzene ring for each isomer resulting in either destructive (DQI) or constructive (CQI) quantum interference, respectively.^3^ This behavior can be observed in all aromatic systems, yet when hetero atoms are included in the conductance path, this can be further altered. One of the best systems to show this is the biphenyl heterocycles series examined by Grace and Alanazy;^4,5^ in this case, the *para*-isomer has a higher conductance than the *meta*-isomer, but in the *para*-anchored series, the conductance was independent of the heterocycle. However, the meta-isomers’ conductances were strongly dependent on the heterocycle, which is further reflected in the variation of Seebeck values.^4^

Such examples of QI have been largely confined to p-orbital-based systems, with few d-orbital-containing systems being reported.^6−9^ When considering QI interactions, they can be divided into three categories:^3^ as Breit–Wigner resonance, in which the energy *E* of an electron passing through the conductor resonates with the backbone state of the molecule, this is responsive to external perturbations, e.g., environment, gating potential, temperature, etc.; *Fano* resonance occurs when *E* coincides with the energy of a bound state located on a pendant group of the conductor backbone, and finally and most pertinent to this work, Mach–Zehnder resonance, in which the electron is able to traverse *n* > 1 paths through the same molecule. In Mach–Zehnder QI interactions with *n* = 2 paths, the conductance of the entire conductor is , where *G*_1_ and *G*_2_ are the conductance values of each path, when *G*_1_ = *G*_2_; this simplifies to *G* = 4*G*_1_. To further investigate the impact of transition metal complexes on the QI of a molecule, a series of compounds containing 2,2′-dipyridyl metal carbonyl complexes was synthesized. This motif was chosen due to its structural similarity to that of the fluorenones and modular synthetic construction in addition to the formation of neutral metal complexes. A thioether anchor was used, owing to its synthetic stability and tendency to act as a midgap anchor group, which should maximize the metal center’s involvement in conductance. Having such an arrangement allows introduction of a transmission metal containing conductance path to be compared to one without, and by varying the metal used, this affords control over the energy levels of the metal center.

## Results and Discussion

**Figure 1:**
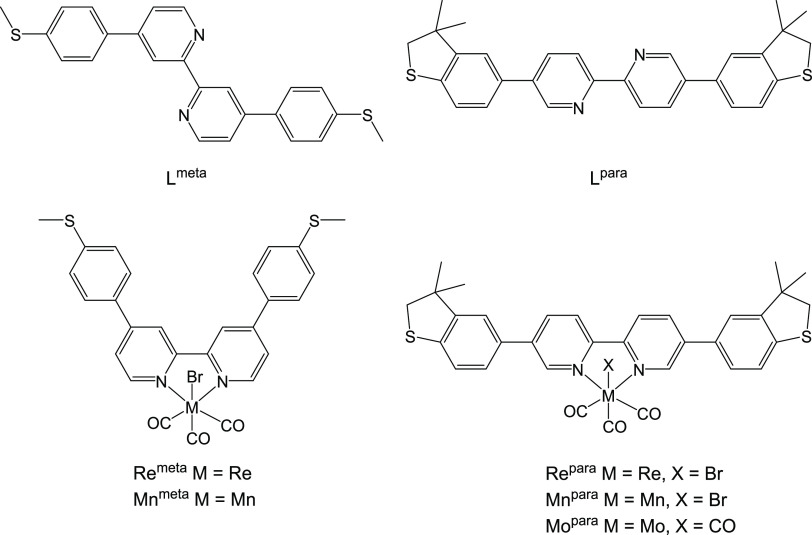
Ligands L^meta^, L^para^, and their corresponding metal complexes (Re^meta^, Re^para^, Mn^meta^, and Mn^para^, Mo^para^) used in this investigation.

The ligand 4,4′-bis(4-(methylthio)phenyl)-2,2′-bipyridine (L^meta^) was prepared by coupling (4-(methylthio)phenyl)boronic acid with 4,4′-dibromo-2,2′-bipyridine via a Suzuki–Miyaura reaction. An analogous reaction was performed using 5,5′-dibromo-2,2′-bipyridine; however, this formed an insoluble material that could not be characterized nor used for further reactions. To improve the solubility of the 5,5′-anchored ligand, 3,3-dimethyl-2,3-dihydrobenzothiophene (DMBT) was used in place of thioanisole to produce 5,5′-bis(3,3-dimethyl-2,3-dihydrobenzothiophen-5-yl)-2,2′-bipyridine (L^para^). The rhenium(I) carbonyl complexes (Re^meta^ and Re^para^) were prepared using the classical approach of heating rhenium(I) pentacarbonyl bromide with the respective ligand in toluene, while the manganese analogues (Mn^meta^ and Mn^para^) were prepared by heating the ligand and manganese(I) pentacarbonyl bromide in diethyl ether. The molybdenum(0) tetracarbonyl complex (Mo^para^) was prepared by irradiating Mo(CO)_6_ in a solution of L^para^ in tetrahydrofuran. Moreover, an analogous reaction was attempted with L^meta^, but the resulting complex proved to be too insoluble to be characterized (Figure 1).

### Molecular Structures

**Figure 2:**
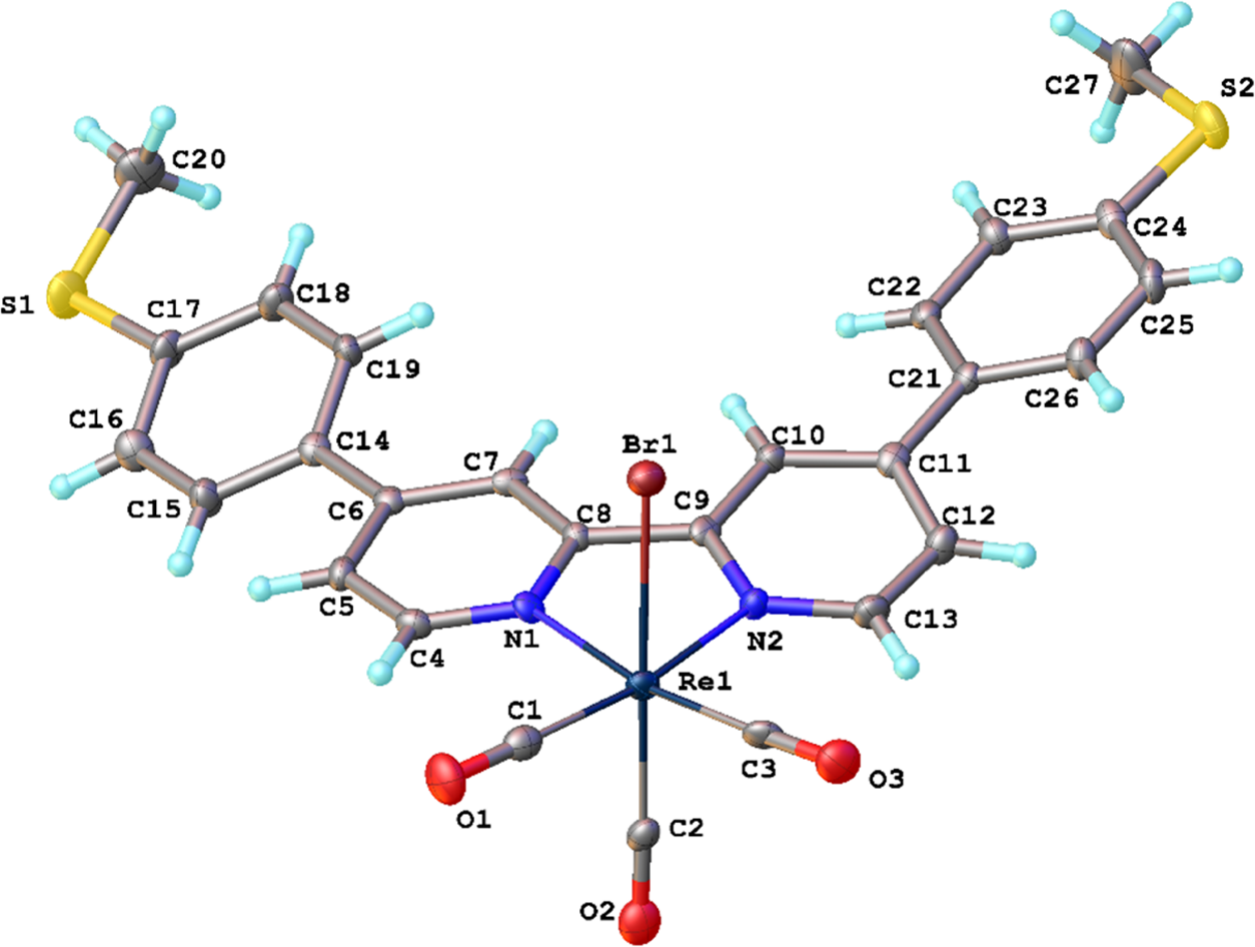
Molecular structure of Re^meta^. Thermal ellipsoids displayed at 50% probability.

**Figure 3:**
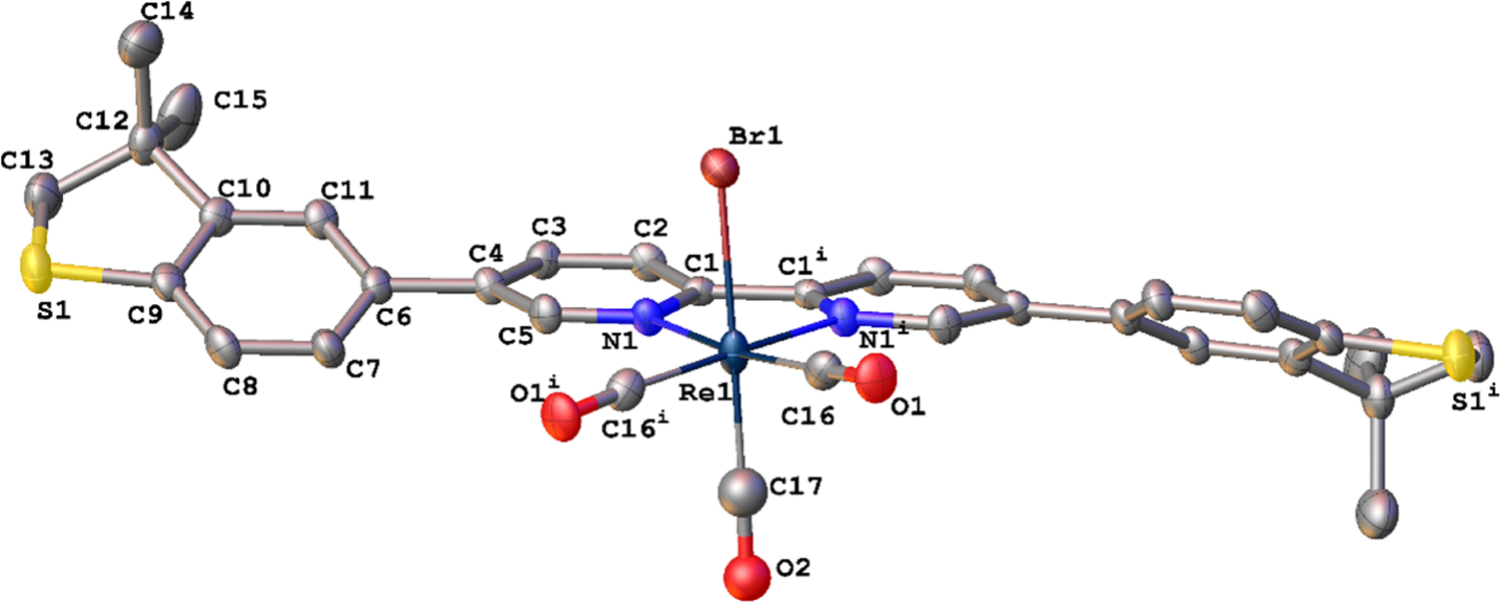
Molecular structure of Re^para^. Thermal ellipsoids displayed at 50% probability.

The crystal structures of L^para^, L^meta^, Re^para^, Re^meta^, and Mn^meta^ were determined by single-crystal X-ray crystallography (CCDC 266928–2266932). Complexes Re^meta^ (Figure 2) and Re^para^ (Figure 3) crystallize as DCM and chloroform di- and monosolvates, respectively. Complex Re^para^ is situated on a 2-fold axis; molecules of both free ligands in crystals are located in the centers of symmetry, i.e., their configurations around central C–C bonds are s-*trans*. Molecules of free ligands are not planar; dihedral angles between mean planes of Py- and Ph-rings are 33.68(4) and 30.1(1)° in L^meta^ and L^para^, respectively. In crystals, molecules of ligands do not form stacking and/or S···S chalcogen interaction bonds and are linked together by a number of weak C–H···π/N/S contacts.

The conformations of both ligands change in coordination to a metal, becoming s-*cis*. The L^meta^ ligand in coordinated form became more planar: in metal complexes, Mn^para^ and Re^meta^, an average dihedral angle between Ph- and Py-rings is equal to 20.4°. In contrast to the structures of free ligands, the π···π interactions are present in all three studied complexes. These interactions combine complexes of L^meta^ into isolated slanted stacks while the different general shape of L^para^ ligands makes Re^para^ complexes form layers. These stacks and layers are linked together and with the solvent molecules by a variety of other weak intermolecular interactions of C–H···X type. Relatively short S···S contacts (of ∼3.5 Å) are present in structures of L^meta^ complexes.

### Conductance

Devices incorporating the structures depicted in Figure 1 were fabricated and characterized using the scanning tunneling microscope-break junction technique (STM-BJ). A detailed description of the technique and the instrumentation used in this contribution can be found elsewhere.^10,11^ In brief, after a regular approach of an Au STM tip to an Au substrate, the feedback loop is disabled and a voltage ramp is applied to the piezoelectric transducer controlling the tip position in the *z* axis to (i) drive the tip into contact with the substrate, thus generating a metallic contact having conductance *G* ≫ *G*_0_ (where *G*_0_ is the quantum of conductance, ), and (ii) withdrawn from the substrate in the presence of the desired molecular wire. During the withdrawal process, the metallic contact is thinned to an atomic point contact and then ruptured. Molecules with appropriate metallophilic termini (e.g., the thioether moieties of the molecules shown in Figure 1) can self-assemble in the resulting nanogap, closing the circuit and generating a single-molecule junction. Further tip withdrawal results in extension of the molecular junction to its full-length state and its consecutive rupture. The process is repeated thousands of times under a DC bias of *V* = 200 mV while the current *I* through the device is continuously monitored with a transimpedance amplifier, recorded with a digital-to-analogue converter, and the conductance is calculated as *G* = *I*/*V*. All data acquired are compiled into statistical histograms and density maps, where the most probable conductance value of the single-molecule junction can be extracted from the contributions at *G* < *G*_0_.

**Figure 4:**
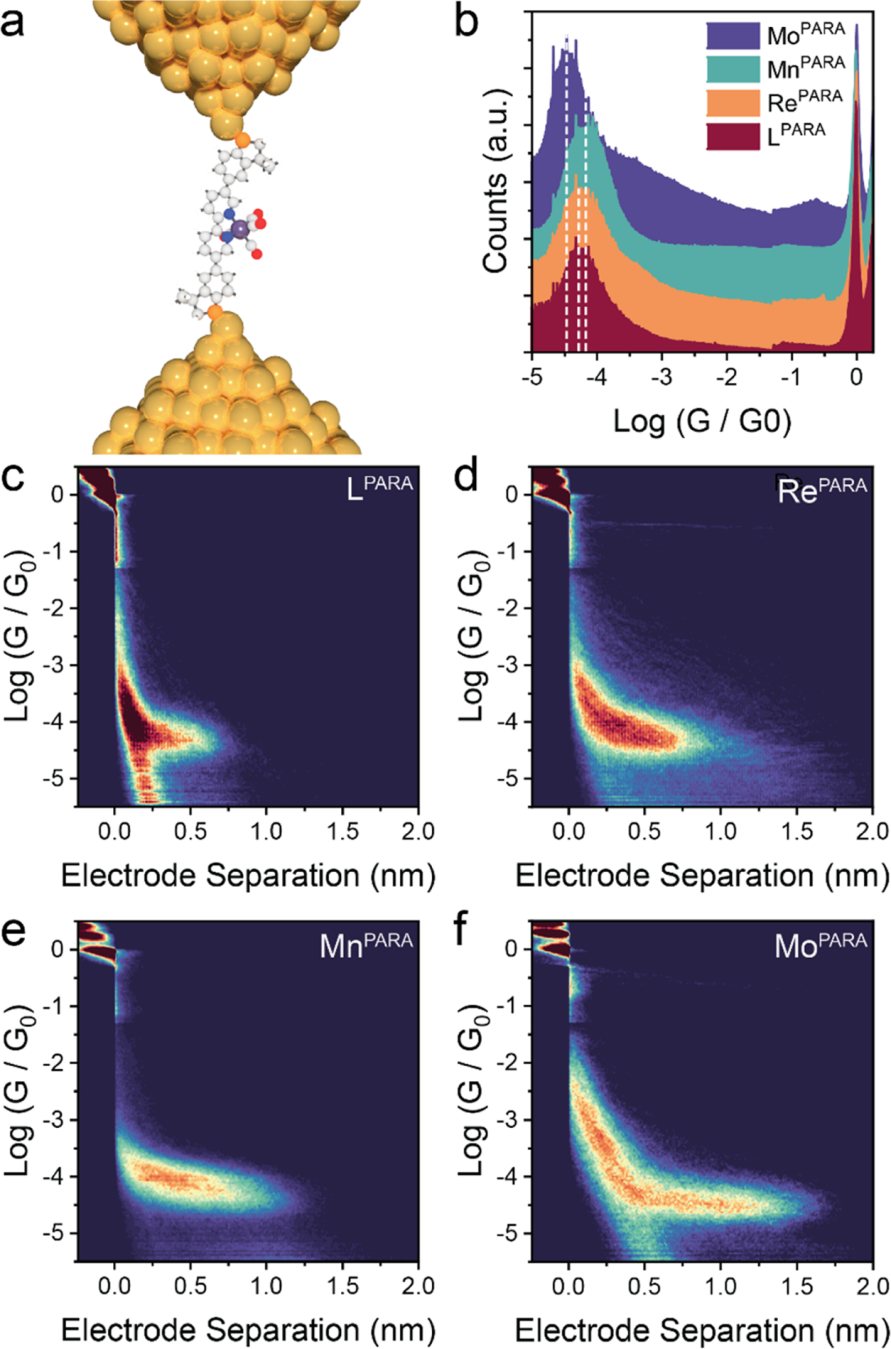
STM-BJ results on the *para* series. (a) Depiction of a single-molecule junction with the compounds used in this study. (b) comparison of the conductance histogram for the *para* molecular wires used in this study. The most probable conductance, extracted by Gaussian fitting of the conductance histogram, is highlighted as a dotted line. (c–f) 2D density maps for the compounds in the *para* series. All data were obtained at 200 mV DC bias, and plots were compiled with no selection from 6356 (L^para^), 4757 (Re^para^), 4605 (Mn^para^), and 4180 (Mo^para^) individual traces.

We started our investigation with the *para* compounds. As can be observed in Figure 4, the free ligand L^para^ and the complex Re^para^ are characterized by very similar conductance values, with distributions centered at 10^–4.3^*G*_0_ in both cases. STM-BJ experiments with Mn^para^ returned a slightly higher charge transport efficiency through the manganese complex, with the conductance distribution in the histogram centered at 10^–4.1^*G*_0_. On the other hand, the molybdenum complex Mo^para^ showed slightly decreased conductance, with the distribution centered at 10^–4.4^*G*_0_ In all cases, the 2D density map shows that junctions are fabricated and stretched through an extended form of the molecular wire, as the high-count area matches molecular length (∼1.4 nm accounting for an electrode snapback of 5 Å).

**Figure 5:**
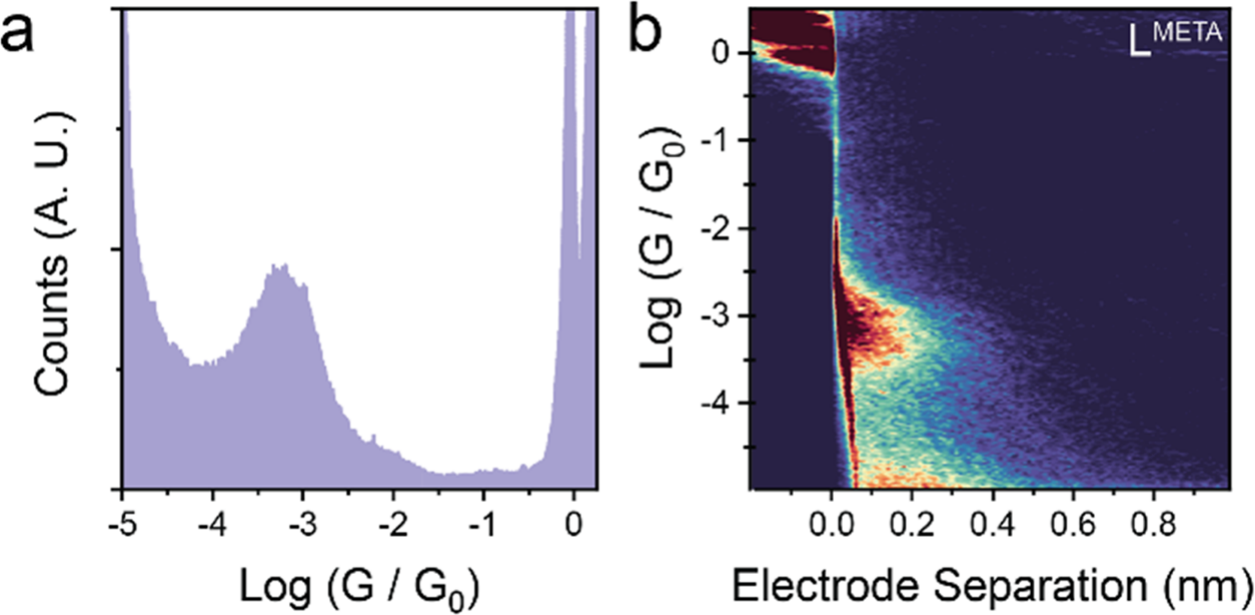
STM-BJ results for L^meta^. (a) Conductance histogram and (b) 2D density map. Data were obtained at 200 mV DC bias, and plots were compiled with no selection from 6206 traces.

While the *para* series highlighted a clear (albeit minor) change in conductance upon complexation, the same behavior could not be evaluated for the *meta* series. For the free ligand, while a single conductance peak centered at 10^–3.2^*G*_0_ can be observed in the histogram, analysis of the conductance traces as a function of electrode separation highlights a very short break-off distance, consistent with transport between the methyl thioether S and one (or both) the pyridyl Ns (Figure 5). Further elongation of the junction results in the conductance trace slowly decaying to the noise level. In the case of the metal complexes, on the other hand, conductance through the molecular wire fails to settle on a plateau, indicative of transport through the extended molecular wire, and no clear peak is observable in the conductance histogram. The absence of the peak we attributed to transport from the methyl thioether to one of the pyridyl N atoms suggests the complexes are robust, with the pyridyl Ns therefore unable to contact the electrodes. Due to the presence of quantum interference features, the *meta* compounds are expected to have inefficient charge transport properties, and their conductance may be too low to be measured with our setup (i.e., *G* < 10^–5.5^*G*_0_).

### Theory

Quantum transport calculations through the *meta-* and *para*-connected metal complexes were performed to understand the experimental results. First, the ground-state geometries of each molecule in the gas phase and in the junctions between two gold electrodes were determined using the SIESTA^12^ implementation of density functional theory (DFT). Then, the ground-state Hamiltonian and overlap matrices were obtained from DFT and combined with the transport code GOLLUM^13^ to calculate transmission coefficient *T*(*E*) for each molecule between the gold electrodes (see theoretical methods for more details). The Landauer formula and obtained *T*(*E*) as shown in the Supporting information (SI) were used to calculate the room-temperature electrical conductance. It is well-known that DFT is less reliable for the prediction of the electronic structure of molecules involving heavy elements such as rhenium. For this reason and because our measurement shows almost the same conductance for complexes with manganese and rhenium, only transport calculations with the manganese complex were performed.

**Figure 6:**
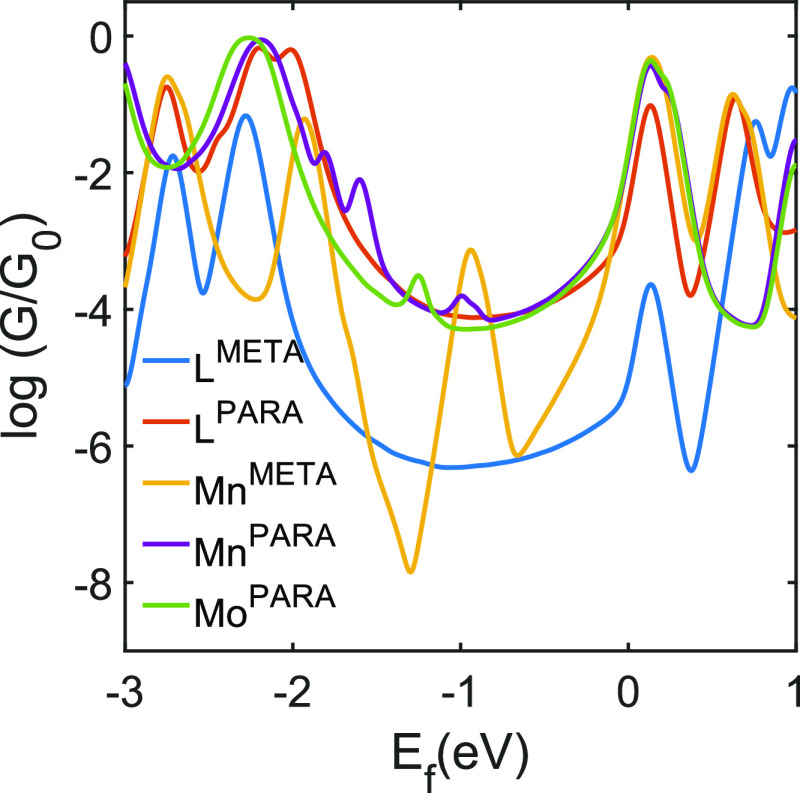
DFT calculated room-temperature electrical conductance for the molecules L^meta^, L^para^, Mn^meta^, Mn^para^, and Mo^para^.

Our calculations show slightly higher electrical conductance for the Mn^para^ and Mo^para^ molecules compared to that of L^para^ around DFT Fermi energy (*E*_F_ = 0 eV in Figure 5), which is in line with the experimental results (Figure 5) and the HOMO–LUMO gap of gas phase molecules (see theory section in the SI); this is attributed to the slight reduction of the HOMO–LUMO gap upon metal coordination, as observed by Ponce et al.^14^ Typically we would expect the *meta*-connected molecules to have lower conductance relative to the *para*-connected analogue,^4,15^ but this contrasts with the observed experimental results for the L^para^ and L^meta^. Indeed, L^meta^ has a conductance feature in the range of 10^–3^*G*_0_ but can be explained by the molecule forming junctions where one electrode is attached to the thiomethyl anchor and the other electrode is attached to the pyridine nitrogen, i.e., effectively behaving as 4-(4-(methylthio)phenyl)pyridine (see Figure S21 in the SI). This is demonstrated in the calculations performed with shorter junctions, as shown in Figures S24 and S25 in the SI. For these configurations, high conductance was obtained in the range of 10^–3^*G*_0_ consistent with the measured value. With this feature explained, we can conclude that, as seen in many previous studies, when contacted via the thioether groups, the *meta*-connected molecules had lower conductance than their *para*-connected analogues.^4,15^ This can be attributed to the DQI near the *E*_F_, which is characterized by antiresonances between the HOMO–LUMO gap in the *meta*-connected molecules (Figure 6). The observed DQI features agree with what is expected from the orbitals (see molecular orbitals of the molecules in SI), and thus, low conductance is predicted in *meta*-connected molecules, as shown in Figure 5. However, unlike with the pure organic molecules analogues, such as bridged biphenyls (e.g., fluorene, fluorenone, etc.),^4,5^ varying the bridging group (i.e., the metal center) had little direct impact on the QI features, with the only impacts being associated with varying the HOMO–LUMO gap. The aforementioned can be attributed to a metal center in this coordination environment being unable to have sufficient orbital mixing to be conjugated with the rest of the molecule; rather, the metal center acts as a localized orbital pendent to the “conductive path”, which means that, regardless of which d-group metal is coordinated to these ligands, the latter will only play a passive role in Mach–Zehnder quantum interference, although the presence of the localized orbital does mean that metals could play a role in Fano resonances.^3^ While such a feature did not impact the measurements of these molecules, Fano resonances can be observed for all the complexes at ca. −1.0 to −1.2 eV *E*–*E*_F_. Given that the energy level of this state is governed by the energies of the metals d-orbitals, it is entirely possible that, through the judicious choice of both the metal and the coligand system, the feature could be shifted close enough to the *E*_F_ to play a role in the conductance of molecules, making that a prime target for further exploration.

## Conclusions

A series of metal complexes based on the M(bpy)(CO)*_n_*X motif were synthesized with thioether-based contact groups in either the *para* or *meta* position as regards the bpy ligand. STM-BJ measurements and DFT calculations showed that metal ion coordination in the *para* system (i.e., peripheral to the conductance path) results in a modest conductance increase relative to the free ligand, with only a negligible difference observed between the rhenium and manganese complexes, explained by the reduced HOMO–LUMO gap. The *meta* system contained a DQI feature; thus, the S···S contacted junction had a conductance below 10^–5.5^*G*_0_, making conductance measurements difficult. However, the comparison to theoretical models revealed that each of the complexes has a Fano resonance that in principle could be modified by a judicial choice of metal and coligands to make such features accessible and that by directly coupling the contact groups to the bpy (i.e., reducing the length of the molecules), the conductance of the *meta* system could be increased to a measurable level.

## Experimental Section

### General Details

NMR spectra were recorded in deuterated solvent solutions on a Varian VNMRS-600 spectrometer and referenced against solvent resonances (^1^H, ^13^C). ASAP data were recorded on a Xevo QTOF (Waters) high-resolution, accurate mass tandem mass spectrometer equipped with an atmospheric pressure gas chromatography (APGC) and atmospheric solids analysis probe (ASAP). Microanalyses were performed by Elemental Microanalysis service, Durham University, U.K. All chemicals were sourced from standard chemical suppliers, except 1-((4-bromophenyl)thio)-2-methylpropan-2-ol,^16^ and 5,5′-dibromo-2,2′-bipyridine,^17^ prepared according to literature methods.

#### 5-Bromo-3,3-Dimethyl-2,3-Ddihydrobenzothiophene (BrDMBT)

1-((4-bromophenyl)thio)-2-methylpropan-2-ol (4.00 g, 15.3 mmol) was added as a solid to a solution containing AlCl_3_ (8.00 g, 60 mmol) in freshly distilled CS_2_ (100 mL) cooled to −78 °C. The solution was stirred at this temperature for 1 h before being allowed to warm to room temperature. Stirring was continued for 16 h before the solvent was removed under vacuum. Water was slowly added to the residue in a well-vented fume hood until no further reaction occurred. Once cooled, the solution was extracted with diethyl ether, the organic layer was then collected, dried over MgSO_4_, and filtered, and the solvent was removed in *vaccuo*. The oil was eluted through a silica plug using hexane, and final purification was carried by kugelrohr distillation at 110 °C (2 mbar). Yield: 2.00 g (54%). ^1^H NMR (500 MHz, CDCl_3_): δ_H_ 1.36 (s, 6H), 3.18 (br s, 1H), 3.09 (s, 2H), 7.04 (d, ^3^*J*_HH_ = 8.2 Hz, 1H), 7.14 (d, ^4^*J*_HH_ = 1.5 Hz, 1H), 7.22 (dd, *J* = 8.2, 1.5 Hz, 1H), consistent with literature data.^16^

#### 2-(3,3-Dimethyl-2,3-Dihydrobenzothiophen-5-yl)-4,4,5,5-Tetramethyl-1,3,2-Dioxaborolane (BPINDMBT)

tBuLi (6.8 mL, 1.9 M, 13.0 mmol) was slowly added to a solution of BrDMBT (3.00 g, 12.4 mmol) in Et_2_O (100 mL) at −78 °C. The solution was stirred for 1 h before adding trimethylborate (1.43 mL, 1.34 g, 13.0 mmol); stirring at −78 °C was continued for 1 h before being allowed to warm to room temperature, and then stirring continued for 16 h. The reaction was quenched with the addition of water and extracted with Et_2_O. The organic layer was collected and dried over MgSO_4_ before the solvent was removed. Pinacol (1.77 g, 15 mmol), MgSO_4_ (8.0 g), and 1,4-dioxane (50 mL) were added to the residue. This suspension was heated to 110 °C for 16 h, after which the solution was cooled and filtered. The solvent was removed from the filtrate in *vaccuo*, producing a colorless oil; purification was achieved by silica chromatography eluted by a solvent gradient from neat hexane to a DCM/Hexane (1:3) solution. The solvent was removed, and the residual oil was dissolved in hexane; upon a slow evaporation, colorless crystals formed, which were used for the next step. Yield: 1.30 g (36%).^1^H NMR (CDCl_3_, 600 MHz) δ_H_ 7.57 (dd, ^3^*J*_HH_ = 7.7 Hz, ^4^*J*_HH_ = 1.9 Hz, 1H, H_b_), 7.45 (d, ^4^*J*_HH_ = 1.9 Hz, 1H, H_a_), 7.19 (dd, ^3^*J*_HH_ = 7.6 Hz, ^4^*J*_HH_ = 1.5 Hz, 1H, H_c_), 3.15 (t, ^4^*J*_HH_ = 1.5 Hz, 2H, H_d_), 1.38 (s, 6H, H_e_), 1.33 (s, 12H, H_f_) ppm. ^13^C{^1^H} NMR (CDCl_3_, 126 MHz) δ_C_ 147.1, 144.8, 134.1, 128.5, 121.8, 83.6, 47.1, 27.4, 24.8 ppm. Acc-MS(ASAP^+^): *m*/*z* 290.1613 [M + H]^+^, calcd for C_16_H_24_O_2_SB *m*/*z* = 290.1626 (|Δ*m*/*z*| = 4.5 ppm).

#### 4,4′-Bis(4-(Methylthio)Phenyl)-2,2′-Bipyridine (L^meta^)

A suspension of 4,4′-dibromo-2,2′-bipyridine (1.00 g, 3.21 mmol), 4-(methylthio)phenyl boronic acid (1.08 g, 6.43 mmol), K_2_CO_3_ (1.77 g, 12.86 mmol) in toluene (40 mL), H_2_O (12 mL), and ethanol (6 mL), was degassed by three freeze–pump–thaw cycles before adding Pd(PPh_3_)_4_ (369 mg, 0.32 mmol). The suspension was heated to reflux for 16 h before the solvent was removed in *vaccuo*. Methanol was added to the residue and filtered, with the precipitate. The solid was washed thoroughly using methanol, followed by DCM. X-ray diffractable crystals were grown by vapor diffusion of *n*-pentane into a chloroform solution. Yield: 0.73 g (57%).^1^H NMR (CDCl_3_, 600 MHz) δ_H_ 8.73 (dd, ^3^*J*_HH_ = 5.1 Hz, ^4^*J*_HH_ = 0.8 Hz, 2H, H_a_), 8.70 (dd, ^4^*J*_HH_ = 1.9 Hz, ^4^*J*_HH_ = 0.8 Hz, 2H, H_c_), 7.73 (d, ^3^*J*_HH_ = 8.0 Hz, 4H, H_d_), 7.54 (dd, ^3^*J*_HH_ = 5.1 Hz, ^4^*J*_HH_ = 1.9 Hz, 2H, H_b_), 7.37 (d, ^3^*J*_HH_ = 8.0 Hz, 4H, H_e_), 2.55 (s, 6H, H_f_) ppm. ^13^C{^1^H} NMR (CDCl_3_, 126 MHz) δ_C_ 156.6, 149.6, 148.6, 140.2, 134.6, 127.4, 126.6, 121.2, 118.7, 15.4 ppm. MS(ASAP^+^): *m*/*z* 401.1148 [M + H]^+^, Anal. Calc. for C_24_H_20_N_2_S_2_·1/3H_2_O: C, 70.90; H, 5.12; N, 6.89%. Found: C, 70.99; H, 4.73; N, 6.83%.

#### 5,5′-Bis(3,3-Dimethyl-2,3-Dihydrobenzo[*b*]Thiophen-5-yl)-2,2′-Bipyridine (L^para^)

A suspension of 5,5′-dibromo-2,2′-bipyridine (1.00 g, 3.21 mmol), BPINDMBT (1.86 g, 6.43 mmol), K_2_CO_3_ (1.77 g, 12.86 mmol) in toluene (40 mL), H_2_O (12 mL), and ethanol (6 mL), was degassed by three freeze–pump–thaw cycles before Pd(PPh_3_)_4_ (369 mg, 0.32 mmol) was added. The suspension was heated to reflux for 16 h before the solvent was removed in *vaccuo*. Dicholoromethane was added to the residue and filtered; the filtrated product was collected and passed through a silica column eluted with a solvent gradient from neat DCM to DCM/acetonitrile (9:1). The solvent was removed, and methanol was added, forming a white precipitate that was collected by filtration. X-ray diffractable crystals were grown by vapor diffusion of pentane into a chloroform solution. Yield: 0.55 g (36%).^1^H NMR (CDCl_3_, 600 MHz) δ_H_ 8.90 (d, ^3^*J*_HH_ = 2.4 Hz, 2H, H_c_), 8.50 (d, ^3^*J*_HH_ = 8.3 Hz, 2H, H_a_), 8.00 (d, ^3^*J*_HH_ = 7.8 Hz, ^4^*J*_HH_ = 2.4 Hz, 2H, H_b_), 7.43 (dd, ^3^*J*_HH_ = 8.0 Hz, ^4^*J*_HH_ = 1.8 Hz, 2H, H_e_), 7.31–7.30 (m, 4H, H_d_ + H_f_), 3.25 (s, 4H, H_g_), 1.45 (s, 12H, H_h_) ppm. ^13^C{^1^H} NMR (CDCl_3_, 126 MHz) δ_C_ 149.1, 147.2, 141.3, 134.8, 134.0, 126.3, 122.9, 121.2, 120.9, 47.3, 27.4 ppm.^1^ MS (ASAP^+^): *m*/*z* 481.165 [M + H]^+^, Anal. calc. for C_30_H_28_N_2_S_2_·1/4H_2_O: C, 74.27; H, 5.92; N, 5.77%. Found: C, 74.35; H, 5.79; N, 5.68%.

#### L^meta^Re(CO)_3_Br (Re^meta^)

Re(CO)_5_Br (100 mg, 0.24 mmol) was added to a solution of L^meta^ (96 mg, 0.24 mmol) in toluene (20 mL). The solution was heated to reflux for 16 h, which was removed in *vaccuo* after the solvent. The yellow residue was triturated with methanol forming a yellow precipitate, which was collected by filtration. Crystals were grown by vapor diffusion of pentane into a THF solution. Yield: 122 mg (68%).^1^H NMR (CDCl_3_, 600 MHz) δ_H_ 9.03 (d, ^3^*J*_HH_ = 5.9 Hz, 2H, H_a_), 8.34 (d, ^4^*J*_HH_ = 1.8 Hz, 2H, H_c_), 7.65 (d, ^3^*J*_HH_ = 8.0 Hz, 4H, H_d_), 7.62 (dd, ^3^*J*_HH_ = 5.8 Hz, ^4^*J*_HH_ = 1.9 Hz, 2H, H_b_), 7.42 (d, ^3^*J*_HH_ = 8.0 Hz, 4H, H_e_), 2.57 (s, 6H, H_f_) ppm. ^13^C{^1^H} NMR (CDCl_3_, 126 MHz) δ_C_ 156.0, 153.3, 150.7, 146.5, 143.2, 131.7, 127.4, 126.5, 124.1, 120.2, 15.0 ppm. Anal. Calcd. for C_27_H_20_BrN_2_O_3_ReS_2_: C, 43.20; H, 2.69; N, 3.73%. Found: C, 43.38; H, 2.68; N, 3.73%.

#### L^para^Re(CO)_3_Br (Re^para^)

Re(CO)_5_Br (100 mg, 0.24 mmol) was added to a solution of L^para^ (118 mg, 0.24 mmol) in toluene (20 mL). The solution was heated to reflux for 16 h, which was removed in *vaccuo* after cooling the solvent. The yellow residue was triturated with methanol, forming a yellow precipitate, which was collected by filtration. Crystals were grown by the slow evaporation of a DCM/MeOH solution. Yield: 149 mg (75%). ^1^H NMR (CDCl_3_, 600 MHz) δ_H_ 9.20 (d, ^4^*J*_HH_ = 2.1 Hz, 2H, H_a_), 8.17 (d, ^3^*J*_HH_ = 8.4 Hz, 2H, H_b_), 8.12 (dd, ^3^*J*_HH_ = 8.4 Hz, ^4^*J*_HH_ = 2.1 Hz, 2H, H_c_), 7.40 (dd, ^3^*J*_HH_ = 8.0 Hz, ^4^*J*_HH_ = 1.9 Hz, 2H, H_f_), 7.35–7.34 (m, 2H, H_e_), 7.28 (d, ^4^*J*_HH_ = 1.9 Hz, 2H, H_d_), 3.27 (d, ^4^*J*_HH_ = 2.3 Hz, 4H, H_g_), 1.46 (d, J = 2.8 Hz, 12H, H_h_) ppm. ^13^C{^1^H} NMR (CDCl_3_, 126 MHz) δ_C_ 153.2, 150.9, 149.8, 143.9, 140.0, 136.0, 130.8, 126.5, 123.4, 122.7, 121.2, 47.4, 27.4 ppm.^2^ MS (MALDI): *m*/*z* 829.7 [M]^+^. Anal. Calcd. for C_33_H_28_BrN_2_O_3_ReS_2_·CH_2_Cl_2_: C, 44.63; H, 3.07; N, 3.04%. Found: C, 44.59; H, 3.30; N, 3.06%.

#### L^meta^Mn(CO)_3_Br (Mn^meta^)

Mn(CO)_5_Br (100 mg, 0.36 mmol) was added to a suspension of L^meta^ (144 mg, 0.36 mmol) in Et_2_O (30 mL) that had been degassed by three freeze–pump–thaw cycles. The suspension was heated to reflux for 4 h in the dark before being cooled, forming a yellow precipitate, which was collected by filtration in the absence of light. Purification was achieved by vapor diffusion of *n*-pentane into a THF solution in the absence of light. Yield: 84 mg (38%). ^1^H NMR (CDCl_3_, 600 MHz) δ_H_ 9.20 (d, ^3^*J*_HH_ = 5.7 Hz, 2H, H_a_), 8.27 (br, 2H, H_c_), 7.64 (br, 2H, H_b_), 7.61 (d, ^3^*J*_HH_ = 5.7 Hz, 4H, H_d_), 7.41 (br, 4H, H_e_), 2.57 (s, 6H, H_f_) ppm.^3^^13^C{^1^H} NMR (CDCl_3_, 126 MHz) δ_C_ 155.9, 153.6, 150.1, 142.6, 132.0, 127.5, 126.5, 123.3, 119.4 ppm. Anal. Calcd. for C_27_H_20_BrMnN_2_O_3_S_2_·1/2C_4_H_8_O: C, 53.14; H, 3.69; N, 4.27%. Found: C, 53.16; H, 3.74; N, 4.20%.

#### L^para^Mn(CO)_3_Br (Mn^para^)

Mn(CO)_5_Br (100 mg, 0.36 mmol) was added to a suspension of L^para^ (175 mg, 0.36 mmol) in Et_2_O (30 mL) that had been degassed by three freeze–pump–thaw cycles. The suspension was heated to reflux for 4 h in the dark before cooling, forming a yellow precipitate, which was collected by filtration in the absence of light. Purification was achieved by the slow evaporation of a DCM/MeOH solution in the absence of light to give a microcrystalline powder. Yield: 75 mg (30%).^1^H NMR (CDCl_3_, 600 MHz) δ_H_ 9.39 (s, 2H, H_a_), 8.10–8.00 (broad, 4H, H_b_+H_c_), 7.41 (d, ^3^*J*_HH_ = 7.5 Hz, 2H, H_e_), 7.34 (d, ^3^*J*_HH_ = 7.5 Hz, 2H, H_f_), 7.30 (s, 2H, H_d_), 3.26 (s, 4H, H_g_), 1.46 (s, 12H, H_h_) ppm.^4^^13^C{^1^H} NMR (CDCl_3_, 126 MHz) δ_C_ 158.3, 153.2, 151.3, 149.7, 143.4, 139.0, 135.6, 131.5, 126.5, 123.3, 121.2, 47.4, 27.4 ppm. Anal. Calcd. for C_33_H_28_BrMnN_2_O_3_S_2_·CH_4_O: C, 55.82; H, 4.41; N, 3.83%. Found: C, 55.95; H, 4.39; N, 3.69%.

#### L^para^Mn(CO)_4_ (Mo^para^)

Mo(CO)_6_ (100 mg, 0.37 mmol) was added to a solution containing L^para^ (181 mg, 0.37 mmol) in THF (10 mL) in a quartz cuvette fitted with a Young’s tap and a magnetic stirrer bar. The solution was degassed via three freeze–pump–thaw cycles. The solution was irradiated using a mercury lamp for 12 h while being stirred; during this process, the solution changed from colorless to deep red. Upon completion, the solvent was removed in *vaccuo*, leaving a red residue that was triturated with methanol to give a red precipitate, which was, in turn, collected by filtration and thoroughly washed with methanol. Final purification was achieved by slow evaporation of a DCM/MeOH solution, forming a red precipitate that was collected by decanting the supernatant liquid and drying the remaining solid under vacuum. Yield: 104 mg (41%).^1^H NMR (CDCl_3_, 600 MHz) δ 9.31 (d, ^4^*J*_HH_ = 2.1 Hz, 2H, H_a_), 8.11 (d, ^3^*J*_HH_ = 8.4 Hz, 2H, H_b_), 8.04 (dd, ^3^*J*_HH_ = 8.4 Hz, ^4^*J*_HH_ = 2.2 Hz, 2H, H_c_), 7.43 (dd, ^3^*J*_HH_ = 8.0 Hz, ^4^*J*_HH_ = 2.0 Hz, 2H, H_e_), 7.34 (d, ^3^*J*_HH_ = 8.0 Hz, 2H, H_f_), 7.29 (d, ^4^*J*_HH_ = 1.9 Hz, 2H, H_d_), 3.26 (s, 4H, H_g_), 1.47 (s, 12H, H_h_) ppm.^5^ IR: 2015, 1922, 1874, 1795 cm^–1^. Anal. Calcd. for C_34_H_28_MoN_2_O_4_S_2_·1/2H_2_O: C, 58.53; H, 4.19; N, 4.02%. Found: C, 58.42; H, 4.12; N, 4.04%.

## Data Availability

Raw STM-BJ data acquired in Liverpool is available on the UoL Data Catalogue under a Creative Commons International license (CC-BY-4.0) at DOI: 10.17638/datacat.liverpool.ac.uk/2244 and at the address: https://datacat.liverpool.ac.uk/id/eprint/2244.

## Supporting Information Available

LMeta (CIF)
LPara (CIF)
MnMeta (CIF)
ReMeta (CIF)
RePara (CIF)
Contains details of synthetic procedures and characterization; NMR spectra; crystallographic data; and details for the theoretical calculations (PDF)

## Supplementary Material

ao3c06555_si_001.cif

ao3c06555_si_002.cif

ao3c06555_si_003.cif

ao3c06555_si_004.cif

ao3c06555_si_005.cif

## Footnotes

1 Compound too insoluble for all carbon signals to be observed.

2 Compound too insoluble for all carbon signals to be observed.

3 Trace paramagnetic impurities resulted in a broadening of NMR signals.

4 Trace paramagnetic impurities resulted in a broadening of NMR signals.

5 Too insoluble for ^13^C{1H} NMR
