# Supplementary material for: Connectivity-Dependent Conductance of 2,2′-Bipyridine-Based Metal Complexes
Source: ACS Omega. 2023 Dec 14;8(51):48958–65. doi: 10.1021/acsomega.3c06555 (PMC10753655; doi:10.1021/acsomega.3c06555)
Supplement: Supplementary file 6 — ao3c06555_si_006.pdf [file ao3c06555_si_006.pdf]

Connectivity dependent conductance of 2,2′-bipyridine based metal complexes

Yahia Chelli[b], Nicolò Ferri[c], Andrea Vezzoli[c], Ross J. Davidson[a,*], James Morris[c], Richard J. Nichols[c], Simon J. Higgins[c], Sara Sangtarash[b], Hatef Sadeghi[b,*], Dmitry S. Yufit[a], Andrew Beeby[a,*]

[a]*Department of Chemistry, Durham University, South Rd, Durham, DH1 3LE, UK*

[b]*School of Engineering, University of Warwick, Library Rd, Coventry CV4 7AL, UK*

[c]*Department of Chemistry, University of Liverpool, Crown St, Liverpool, L69 7ZD, UK*

*To whom correspondence should be addressed. Email: Ross Davidson (ross.davidson@durham.ac.uk), Hatef Sadeghi (hatef.sadeghi@warwick.ac.uk), and Andrew Beeby (andrew.beeby@durham.ac.uk).

Table of Contents

S1. NMR spectra of reported compounds	S3
S2. Crystallography	S11
S3. Theory	S15
S4. References	S23

S1. NMR spectra of reported compounds

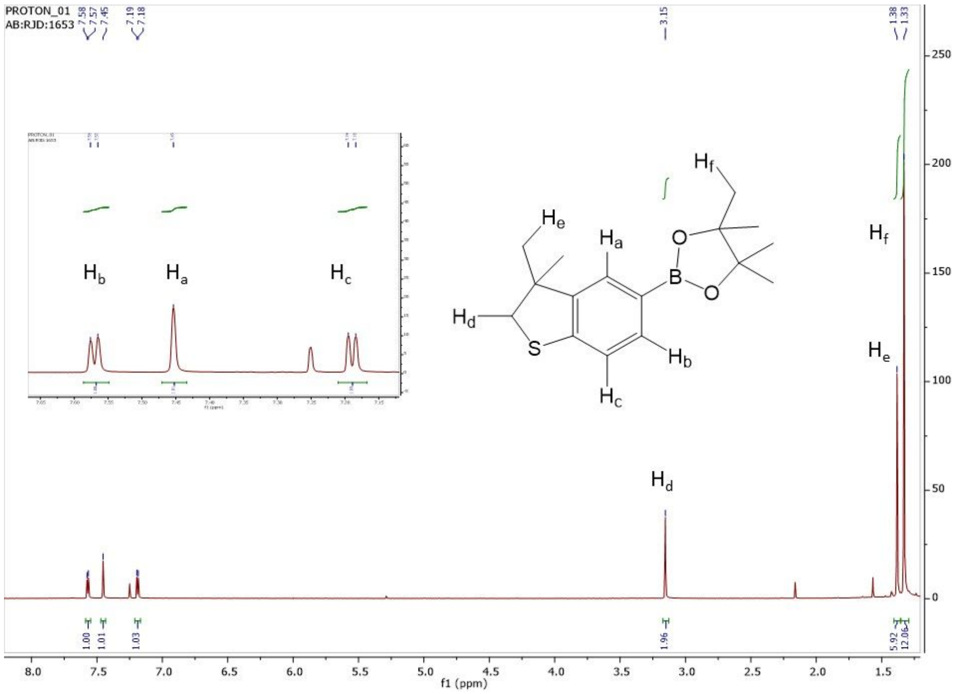

Figure S1. ^{1}H NMR spectrum BPINDMBT recorded in $CDCl_3$.

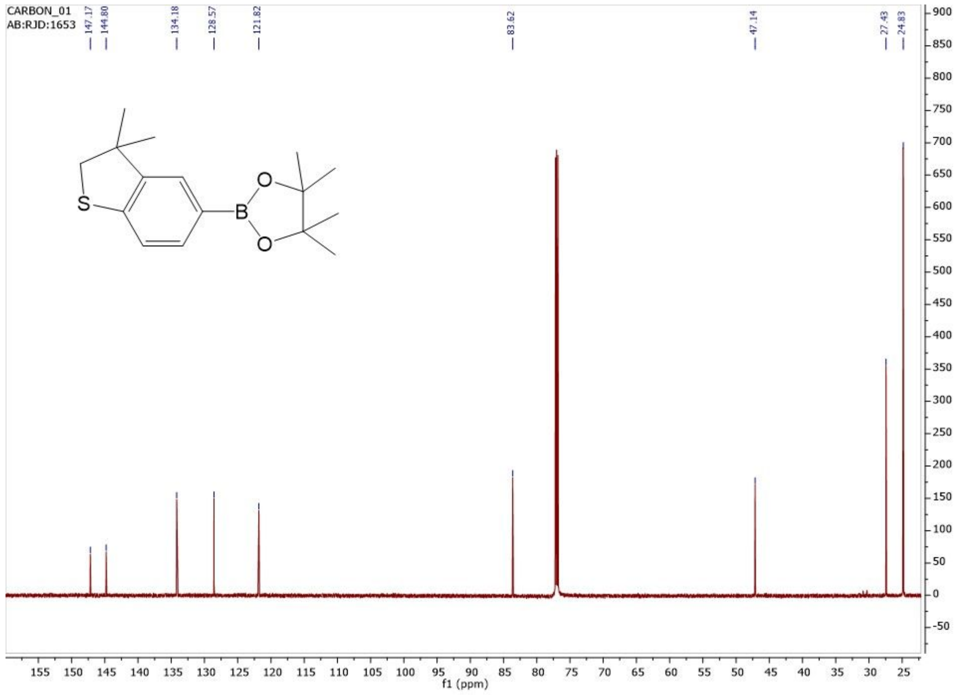

Figure S2. $^{13}C\{^{1}H\}$ NMR spectrum BPINDMBT recorded in $CDCl_3$.

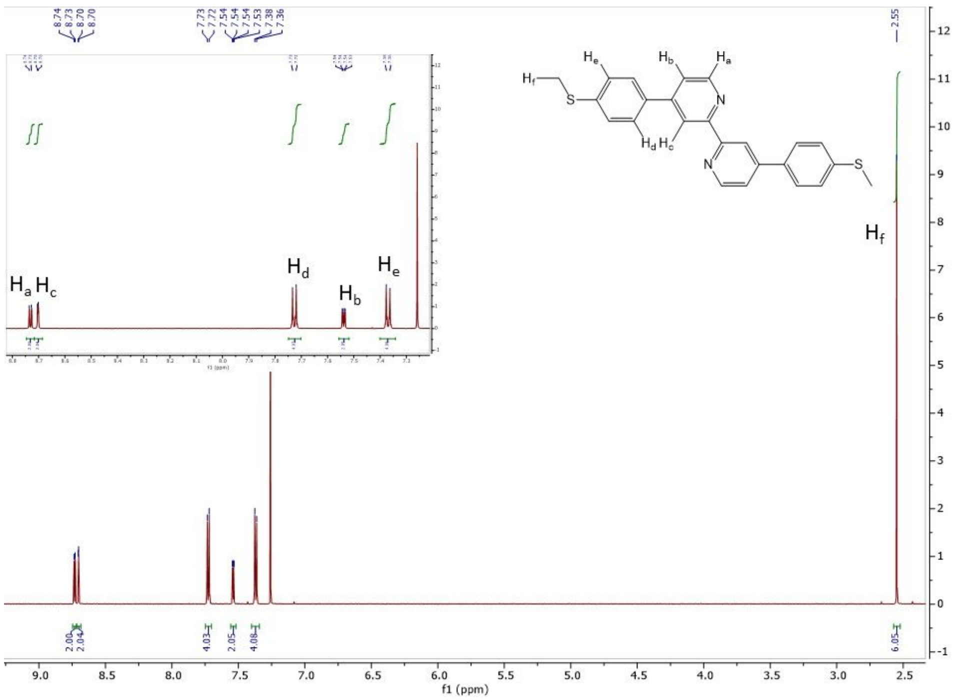

Figure S3. ^{1}H NMR spectrum L^{meta} recorded in $CDCl_3$.

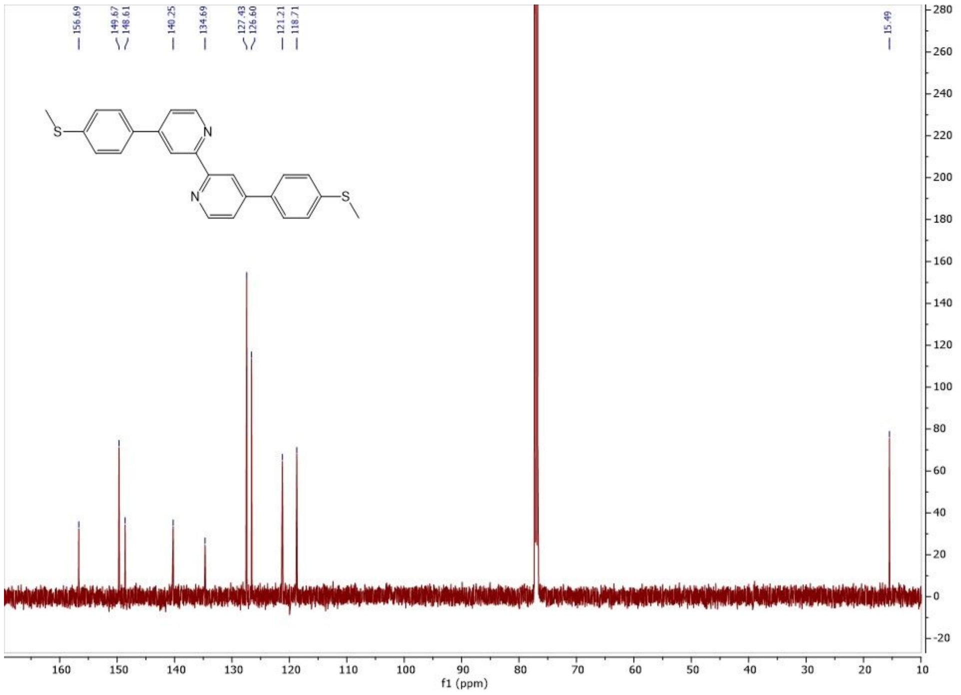

Figure S4. $^{13}C\{^{1}H\}$ NMR spectrum L^{meta} recorded in $CDCl_3$.

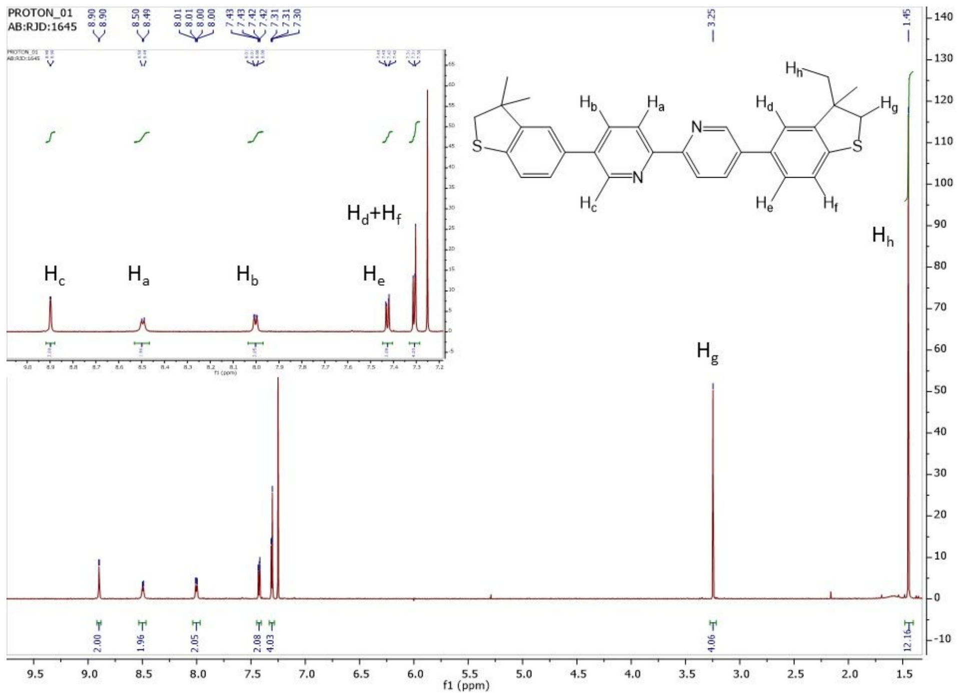

Figure S5. ^{1}H NMR spectrum L[para] recorded in $CDCl_3$.

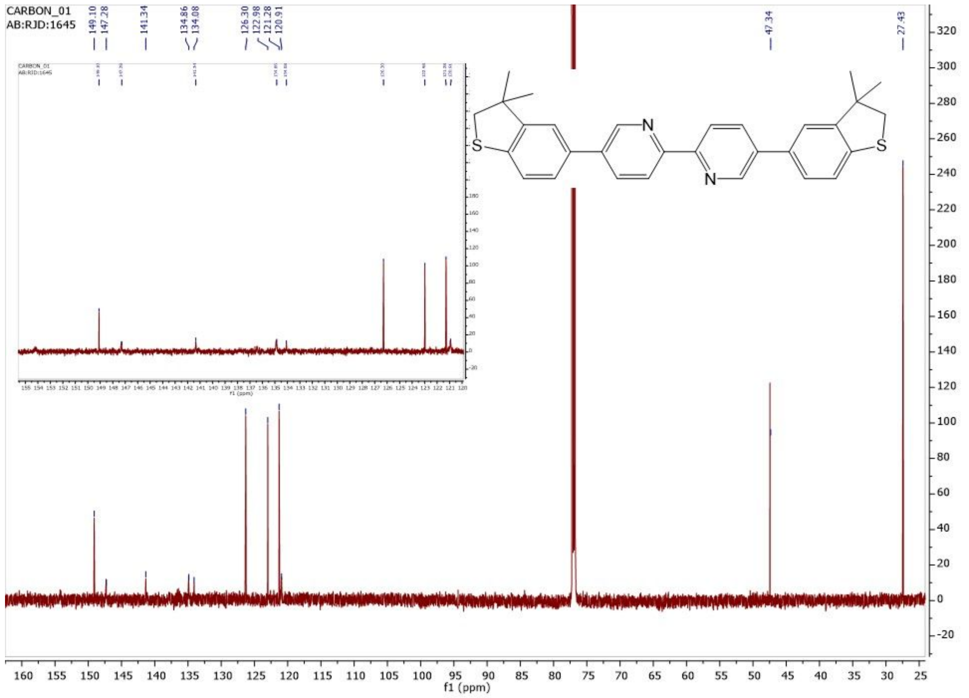

Figure S6. $^{13}C\{^1H\}$ NMR spectrum L[para] recorded in $CDCl_3$.

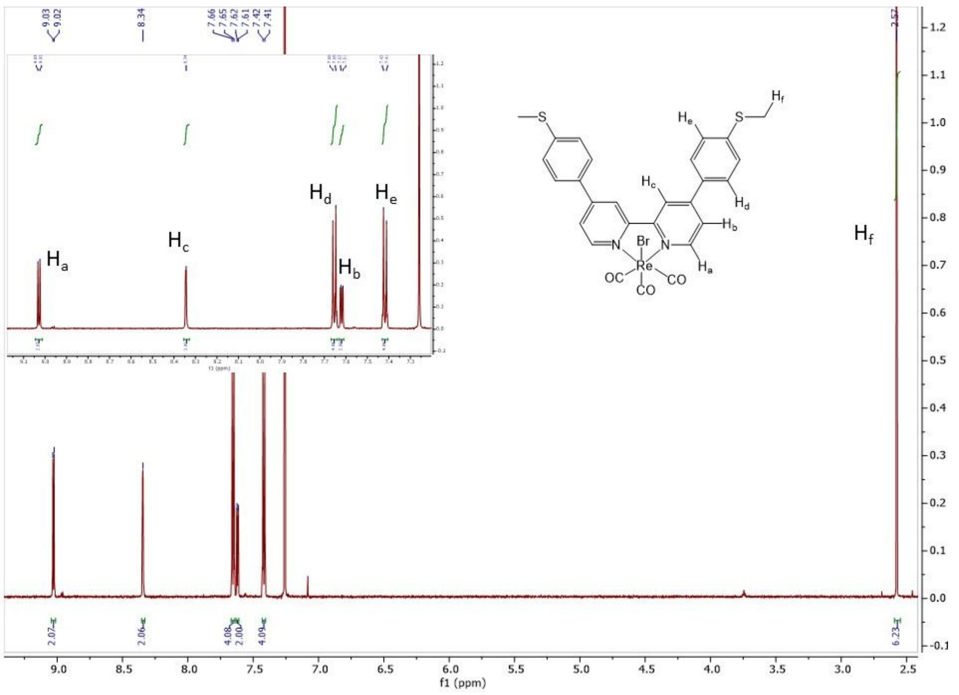

Figure S7. ^{1}H NMR spectrum Re^{meta} recorded in $CDCl_3$.

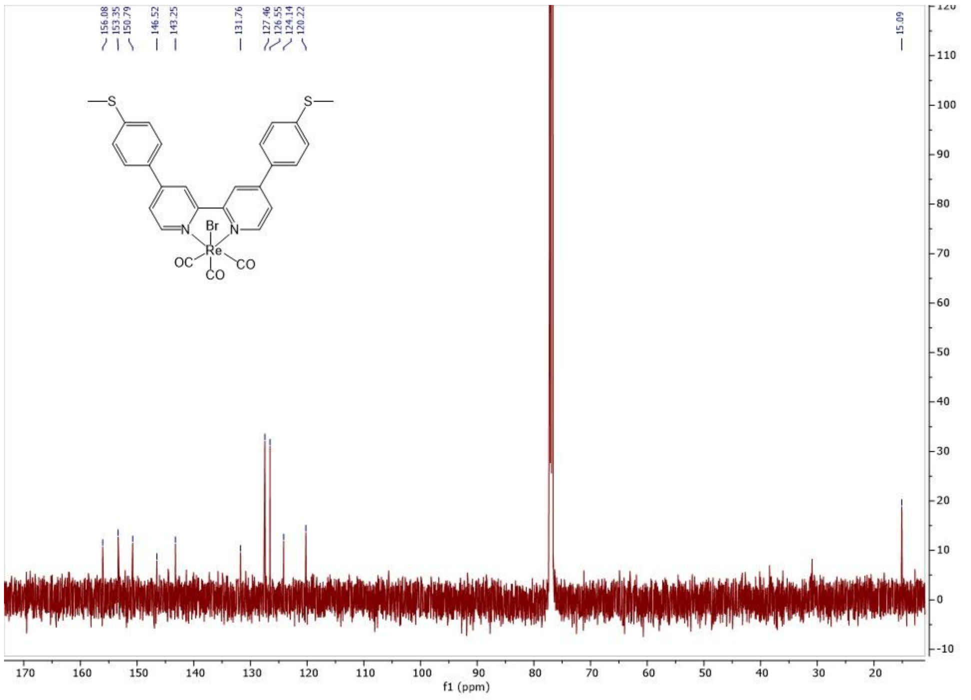

Figure S8. $^{13}C\{^{1}H\}$ NMR spectrum Re^{meta} recorded in $CDCl_3$.

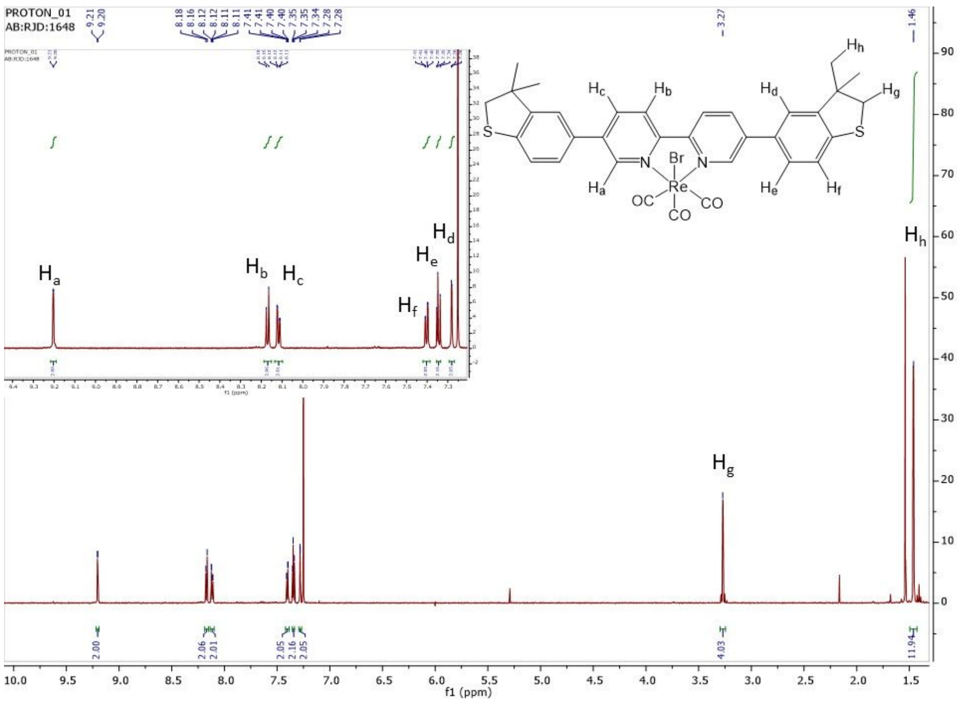

Figure S9. ^{1}H NMR spectrum Re^{para} recorded in $CDCl_3$.

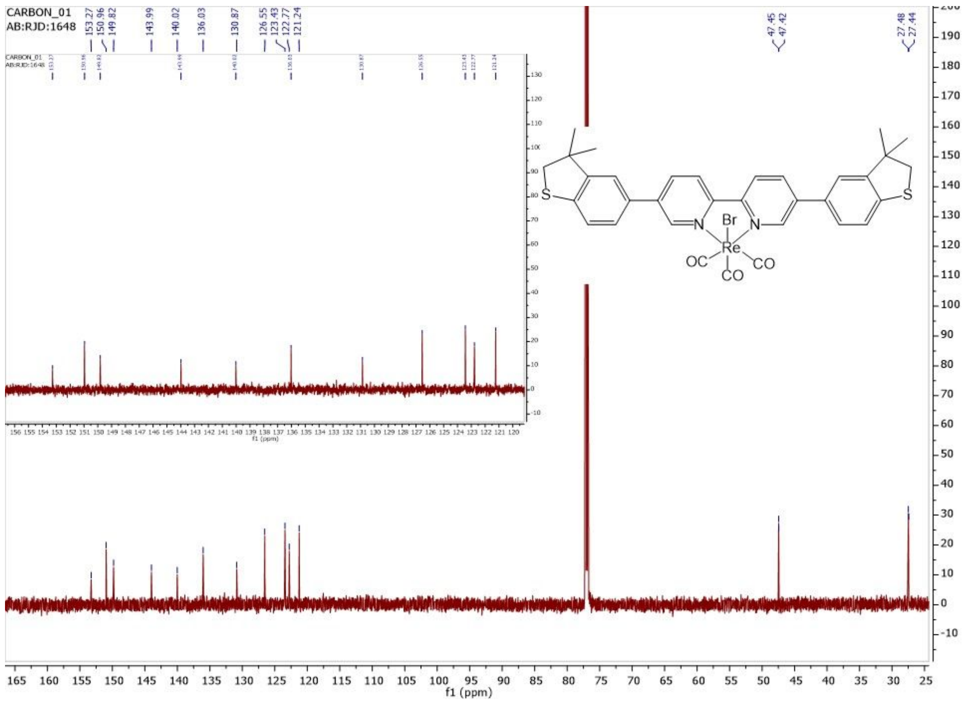

Figure S10. $^{13}C\{^{1}H\}$ NMR spectrum Re^{para} recorded in $CDCl_3$.

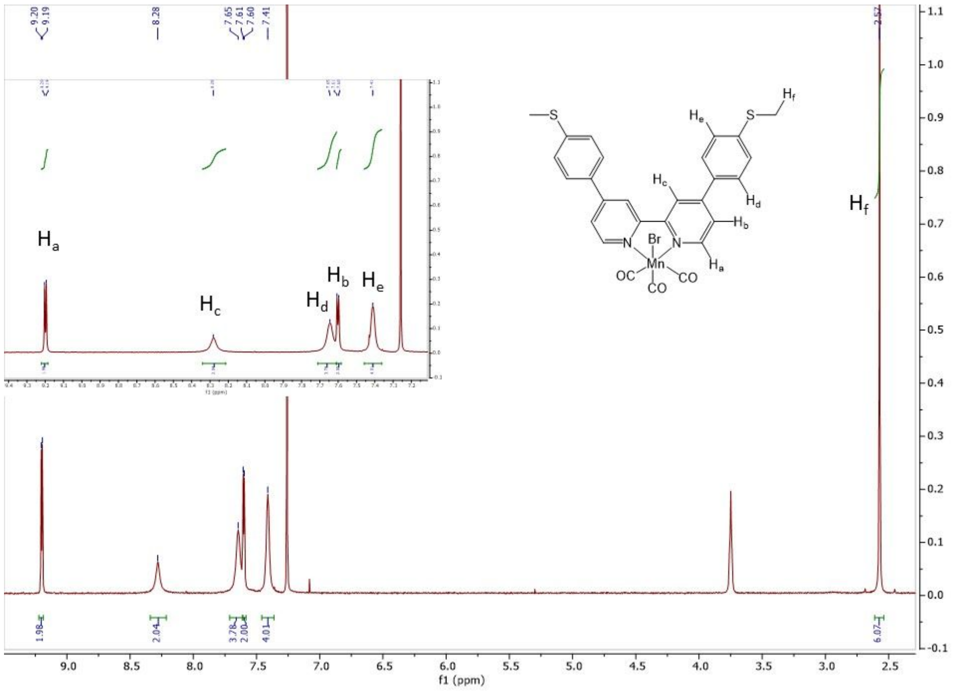

Figure S11. ^{1}H NMR spectrum Mn^{meta} recorded in $CDCl_3$.

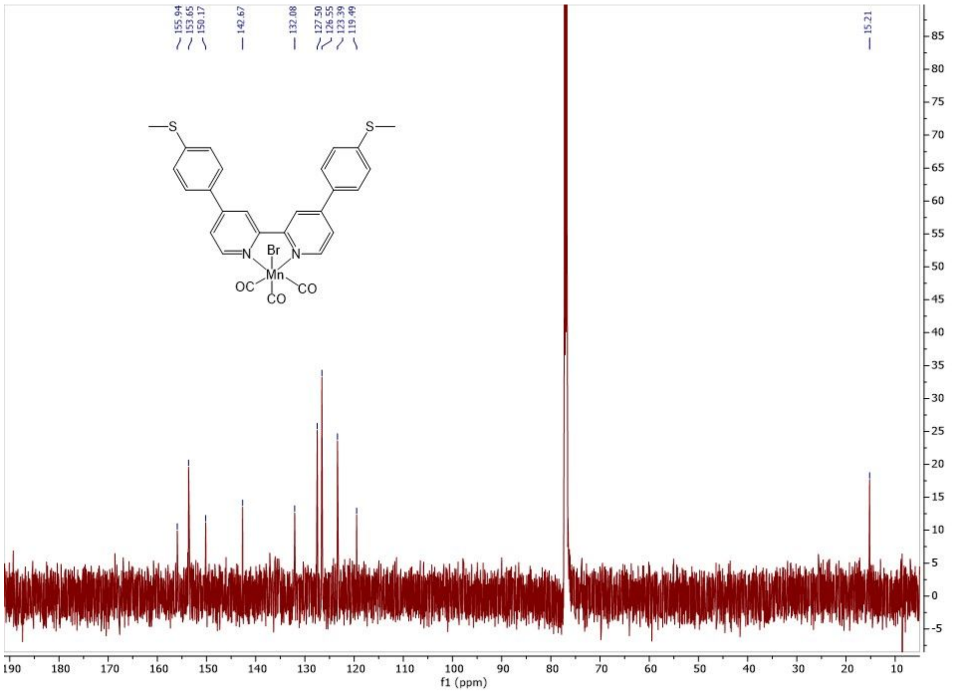

Figure S12. $^{13}C\{^{1}H\}$ NMR spectrum Mn^{meta} recorded in $CDCl_3$.

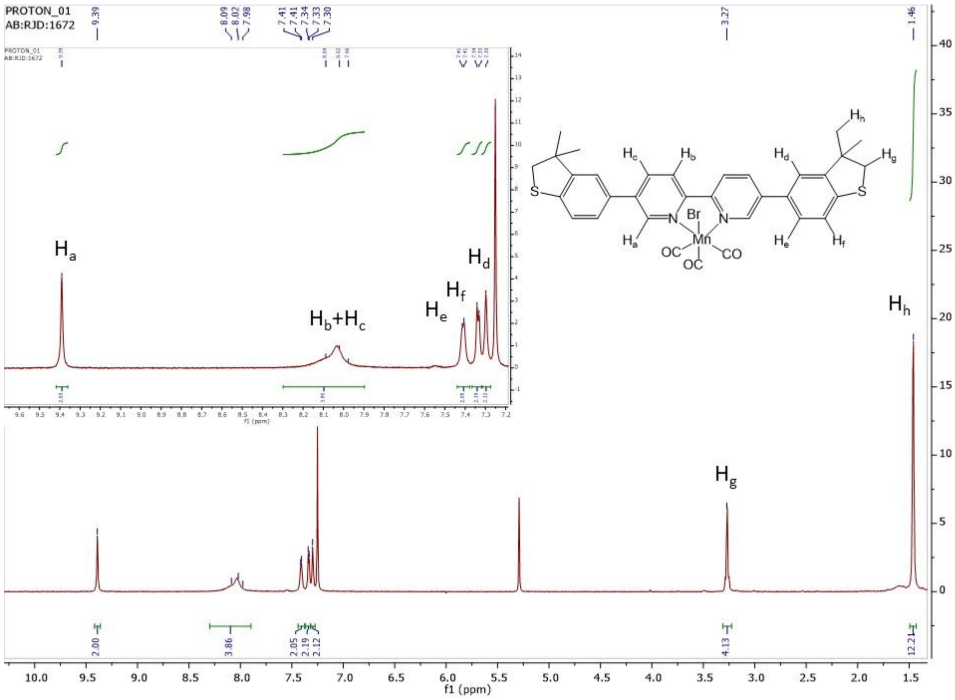

Figure S13. ^{1}H NMR spectrum Mn^{para} recorded in $CDCl_3$.

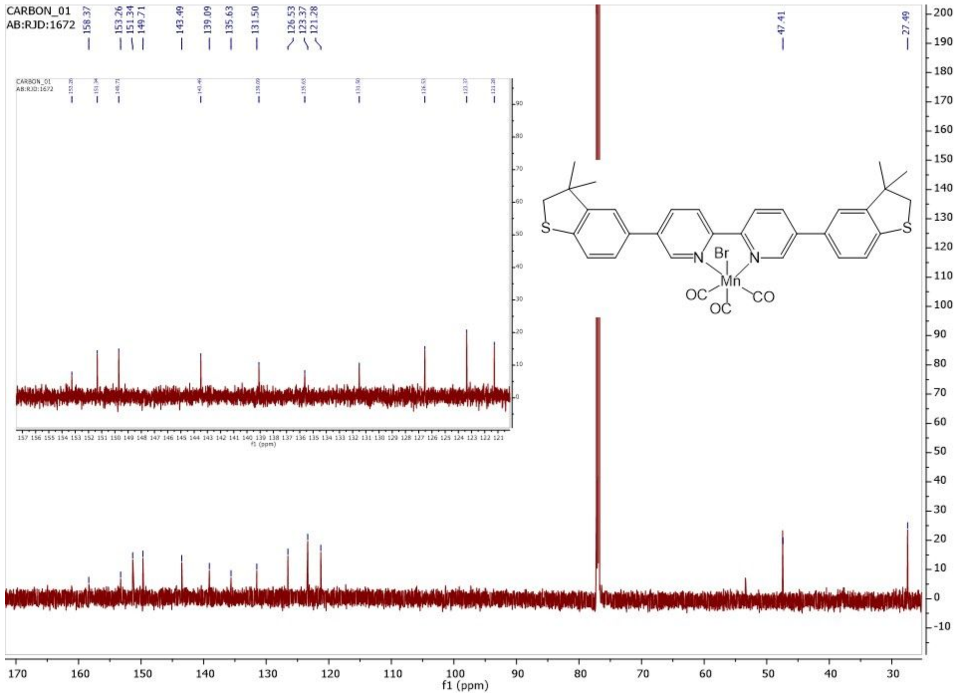

Figure S14. $^{13}C\{^{1}H\}$ NMR spectrum Mn^{para} recorded in $CDCl_3$.

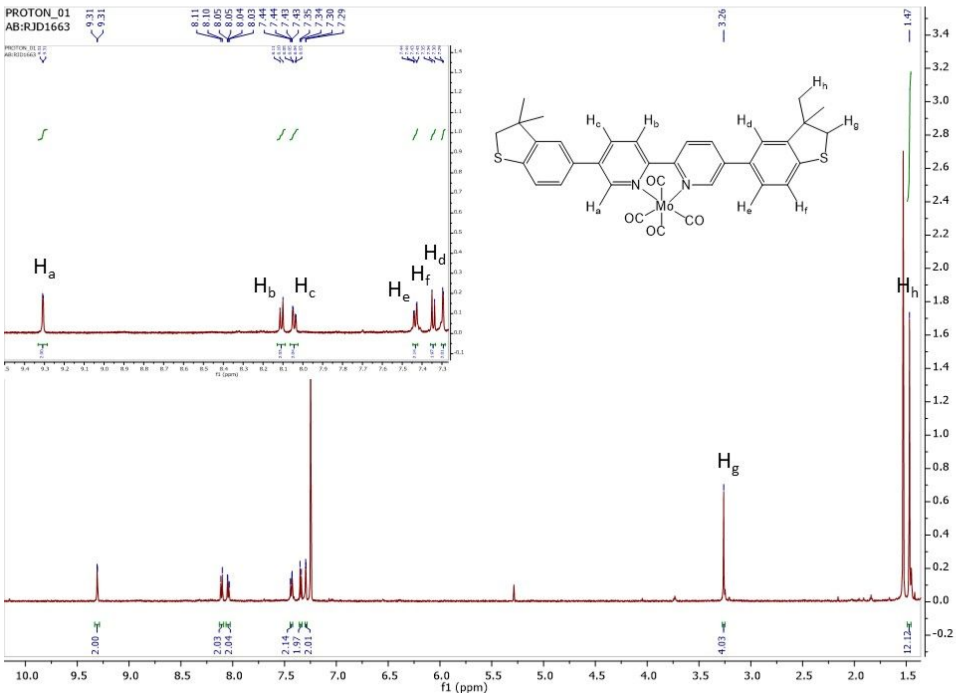

Figure S15. ^{1}H-NMR spectrum Mo^{para} recorded in $CDCl_3$.

S2. Crystallographic data

The X-ray single crystal data have been collected using λMoKα radiation (λ =0.71073Å) on Bruker D8Venture 3-circle diffractometers (Photon100 CMOS detector, IμS-microsource, focusing mirrors (compounds L^{Para} and Re^{Para}))/(Photon III MM C14 CPAD detector, IμS-III-microsource, focusing mirrors (all other compounds)) equipped with a Cryostream (Oxford Cryosystems) open-flow nitrogen cryostats at 120.0(2)K. All structures were solved by direct method and refined by full-matrix least squares on F^2 for all data using Olex2[3] and SHELXTL[4] software. All non-hydrogen atoms were refined in anisotropic approximation, hydrogen atoms in structure L^{Meta} were refined isotropically, the hydrogen atoms in all other structures were placed in the calculated positions and refined in riding mode. Disordered C and O atoms in structure Re^{Para} were refined isotropically with fixed SOF=0.5. Crystal data and parameters of refinement are listed in Tables S1-2. Crystallographic data for the structure have been deposited with the Cambridge Crystallographic Data Centre as supplementary publication CCDC 266928 – 2266932.

Table S1. Crystal data and structure refinement for structures L^{meta}, L^{para} and Re^{meta}.

Identification code	L^{meta}	L^{para}	Re^{meta}
Empirical formula	$C_{24}H_{20}N_2S_2$	$C_{30}H_{28}N_2S_2$	$C_{27}H_{20}BrN_2O_3ReS_2$
Formula weight	400.54	480.66	750.68
Temperature/K	120.0	120.0	120.0
Crystal system	monoclinic	triclinic	monoclinic
Space group	$P2_1/n$	P-1	$P2_1/c$
a/Å	6.7319(3)	5.4765(7)	7.0628(3)
b/Å	8.1059(3)	7.1474(9)	22.5728(10)
c/Å	17.9837(8)	16.398(2)	15.8769(7)
α/°	90	101.648(5)	90
β/°	99.5491(15)	92.645(6)	93.5042(16)
γ/°	90	108.484(6)	90
Volume/Å^3	967.74(7)	592.00(13)	2526.48(19)
Z	2	1	4
ρ_{calc}g/cm^3	1.375	1.348	1.974
μ/mm^{-1}	0.288	0.248	6.590
F(000)	420.0	254.0	1448.0
Crystal size/mm^3	0.32 × 0.09 × 0.06	0.28 × 0.11 × 0.015	0.19 × 0.015 × 0.01
Radiation	MoKα (λ = 0.71073)	MoKα (λ = 0.71073)	MoKα (λ = 0.71073)
2Θ range for data collection/°	4.594 to 59.994	5.108 to 55.996	4.43 to 59.996
Index ranges	$-9 \leq h \leq 9, -11 \leq k \leq 11, -25 \leq l \leq 25$	$-7 \leq h \leq 7, -9 \leq k \leq 9, -21 \leq l \leq 21$	$-9 \leq h \leq 9, -31 \leq k \leq 31, -22 \leq l \leq 22$
Reflections collected	22513	11572	56628
Independent reflections	2818 [R_{int} = 0.0522, R_{sigma} = 0.0281]	2857 [R_{int} = 0.0696, R_{sigma} = 0.0843]	7359 [R_{int} = 0.0863, R_{sigma} = 0.0557]
Data/restraints/parameters	2818/0/167	2857/0/156	7359/24/327
Goodness-of-fit on F^2	1.085	1.175	1.212
Final R indexes [I>=2σ (I)]	R_1 = 0.0347, wR_2 = 0.0888	R_1 = 0.0671, wR_2 = 0.1530	R_1 = 0.0595, wR_2 = 0.0903
Final R indexes [all data]	R_1 = 0.0379, wR_2 = 0.0909	R_1 = 0.0998, wR_2 = 0.1624	R_1 = 0.0777, wR_2 = 0.0945

Table S2. Crystal data and structure refinement for structures Re^{para} and Mn^{meta}.

Identification code	17srv340	21srv303
Identification code	Re^{para}	Mn^{meta}
Empirical formula	$C_{33}H_{28}BrN_2O_3ReS_2 \times 2CH_2Cl_2$	$C_{28}H_{21}BrCl_3MnN_2O_3S_2$
Formula weight	1000.65	738.79
Temperature/K	120.0	120.0
Crystal system	monoclinic	triclinic
Space group	C2/c	P-1
a/Å	30.488(3)	10.6681(3)
b/Å	9.5914(10)	11.1275(3)
c/Å	12.4229(14)	13.3642(4)
α/°	90	78.6860(10)
β/°	91.433(3)	72.4450(10)
γ/°	90	82.6620(10)
Volume/$Å^3$	3631.6(7)	1479.18(7)
Z	4	2
$\rho_{calc}g/cm^3$	1.830	1.659
μ/mm^{-1}	4.894	2.243
F(000)	1960.0	740.0
Crystal size/mm^3	0.23 × 0.11 × 0.01	0.21 × 0.09 × 0.05
Radiation	MoKα (λ = 0.71073)	MoKα (λ = 0.71073)
2Θ range for data collection/°	5.346 to 55.982	4.016 to 59.994
Index ranges	$-40 \le h \le 40$, $-12 \le k \le 12$, $-16 \le l \le 16$	$-15 \le h \le 15$, $-15 \le k \le 15$, $-18 \le l \le 18$
Reflections collected	25206	35735
Independent reflections	4388 [R_{int} = 0.0798, R_{sigma} = 0.0704]	8569 [R_{int} = 0.0332, R_{sigma} = 0.0290]
Data/restraints/parameters	4388/2/223	8569/0/363
Goodness-of-fit on F^2	1.026	1.034
Final R indexes [I>=2σ (I)]	R_1 = 0.0422, wR_2 = 0.0859	R_1 = 0.0293, wR_2 = 0.0739
Final R indexes [all data]	R_1 = 0.0676, wR_2 = 0.0943	R_1 = 0.0349, wR_2 = 0.0766

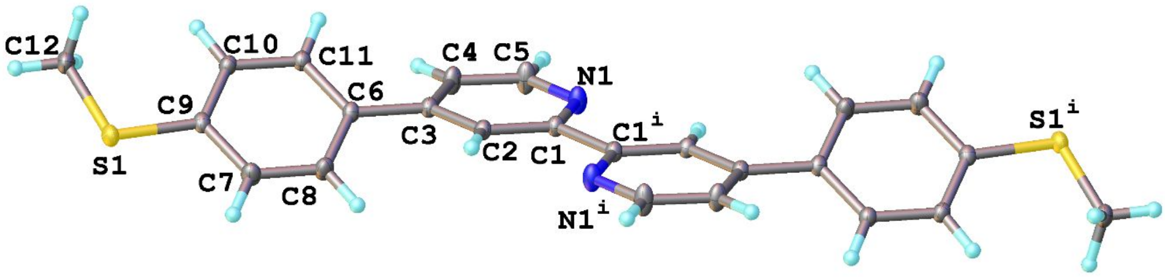

Figure S16. Molecular structure of L^{meta}. Thermal ellipsoids displayed at 50% probability.

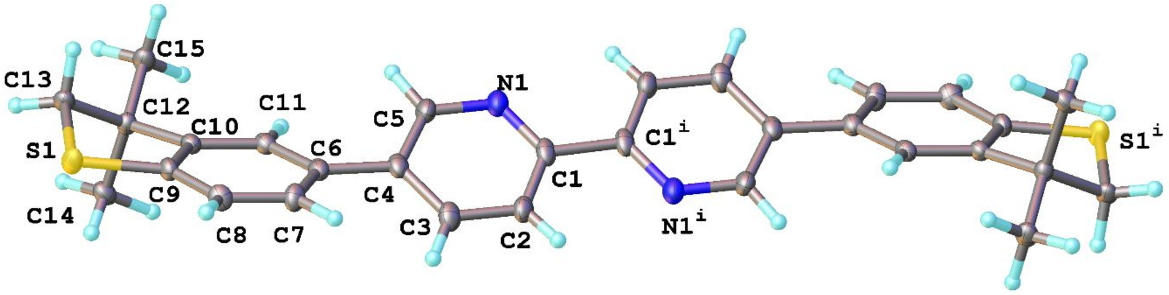

Figure S17. Molecular structure of L^{para}. Thermal ellipsoids displayed at 50% probability.

S3. Theory

Frontier Orbital comparison

Due to the complexity of modelling a rhenium complexes (Re^{meta} and Re^{para}) in a molecular junction the comparison was made using analogous manganese complexes (Mn^{meta} and Mn^{para}) shown in figures S18 and S19 are a comparison of the orbital distributions and energies of the rhenium and manganese complexes in the gas phase. The DFT calculations were performed on Gaussian09[5] using B3LYP, for the manganese complexes a 6-31G(d) basis set was employed for all atoms while for the rhenium complexes a 6-31G(d)/LANL2DZ basis was used. In each case the relative atomic contributions to the frontier orbitals we very similar, with the HOMO localised to the metal centre and the LUMO to the ligand (L^{meta} or L^{para}). There were modest differences observed for the energy levels of the LUMOs, however, the HOMO energies displayed only a negligible difference between the rhenium complexes and their manganese analogues. Taking these factors into account it is reasonable say that the more advanced calculations on the manganese complexes will offer insight into the behaviour of the rhenium complexes.

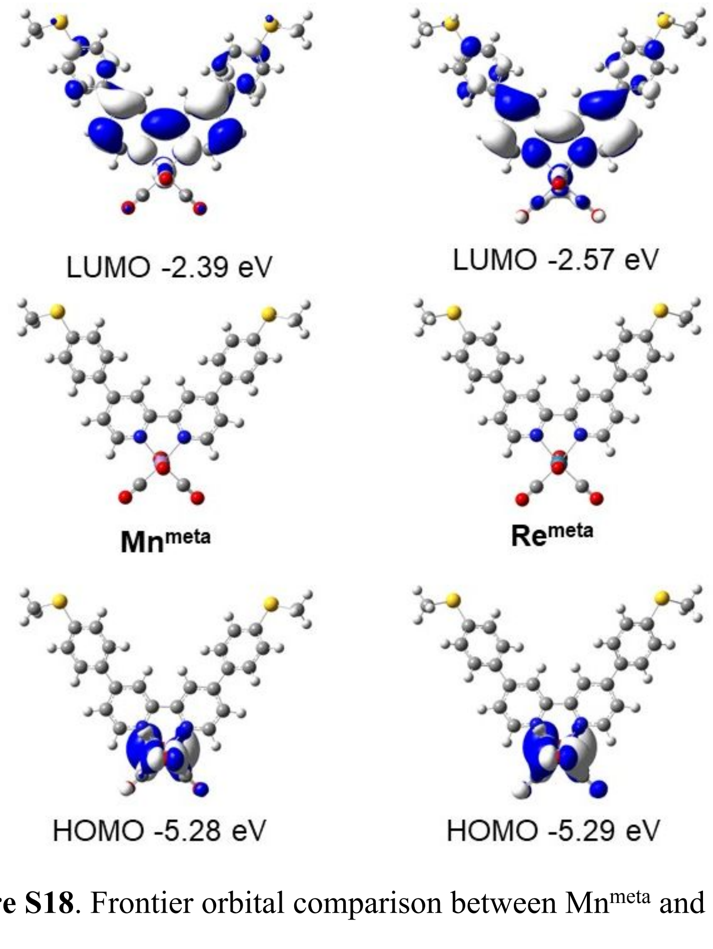

Figure S18. Frontier orbital comparison between Mn^{meta} and Re^{meta}.

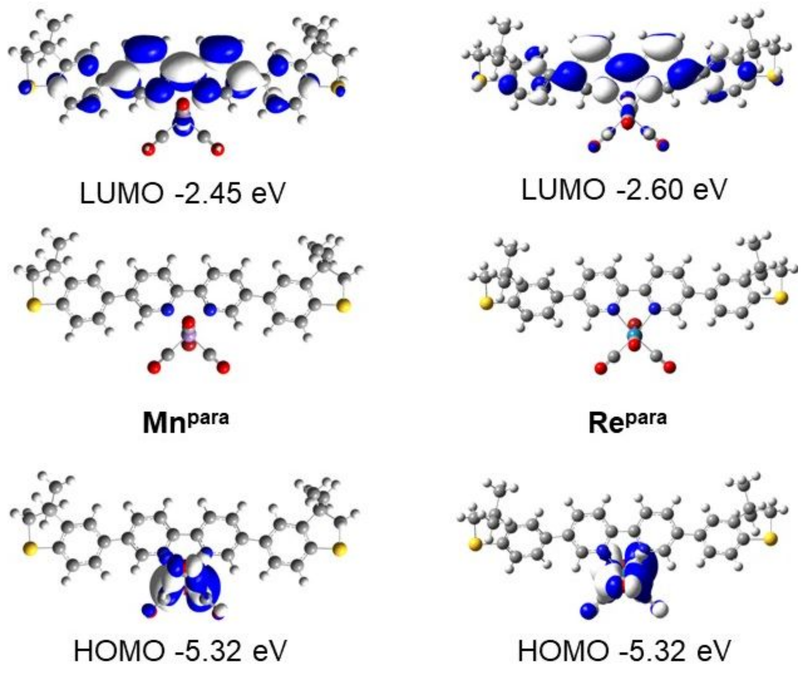

Figure S19. Frontier orbital comparison between Mn^{para} and Re^{para}.

Gold|Molecule|Gold junction geometry

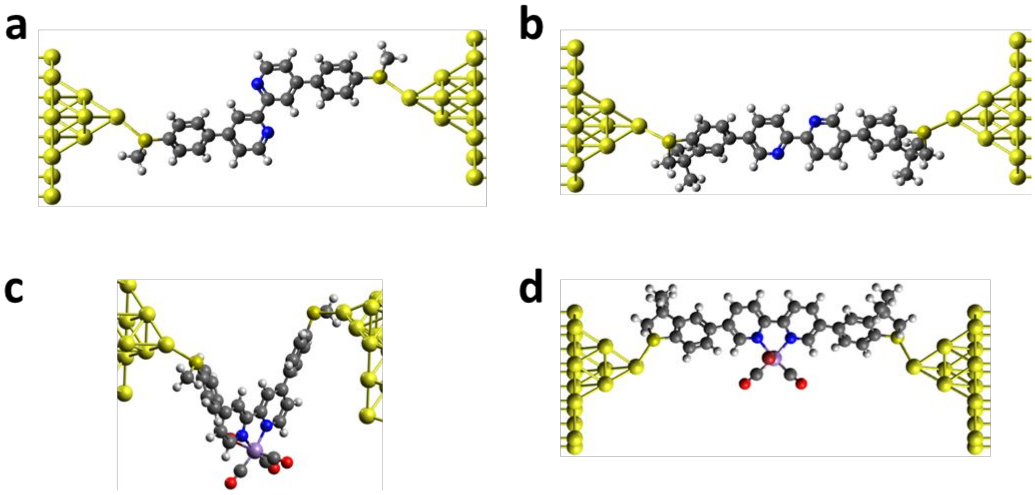

Figure S20. Structure of molecules between two gold electrodes contacting via the thiomethyl groups. (a) L^{meta}, (b) L^{para}, (c) Mn^{meta} and (d) Mn^{para}.

As highlighted in the conductance section, the dominant conductance feature of L^{meta} corresponds to the junction where the pyridyl ring and thiomethyl contact the gold electrode (see Figure S18), denotate as $L^{meta}2$ orientation.

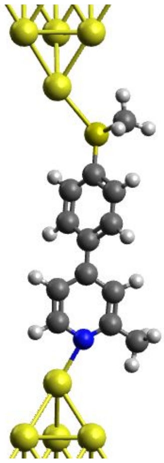

Figure S21. Molecular structure of a L^{meta} fragment between gold electrodes contacting by the pyridine and thiomethyl groups (L^{meta}(py-SMe)).

Orbital Diagrams

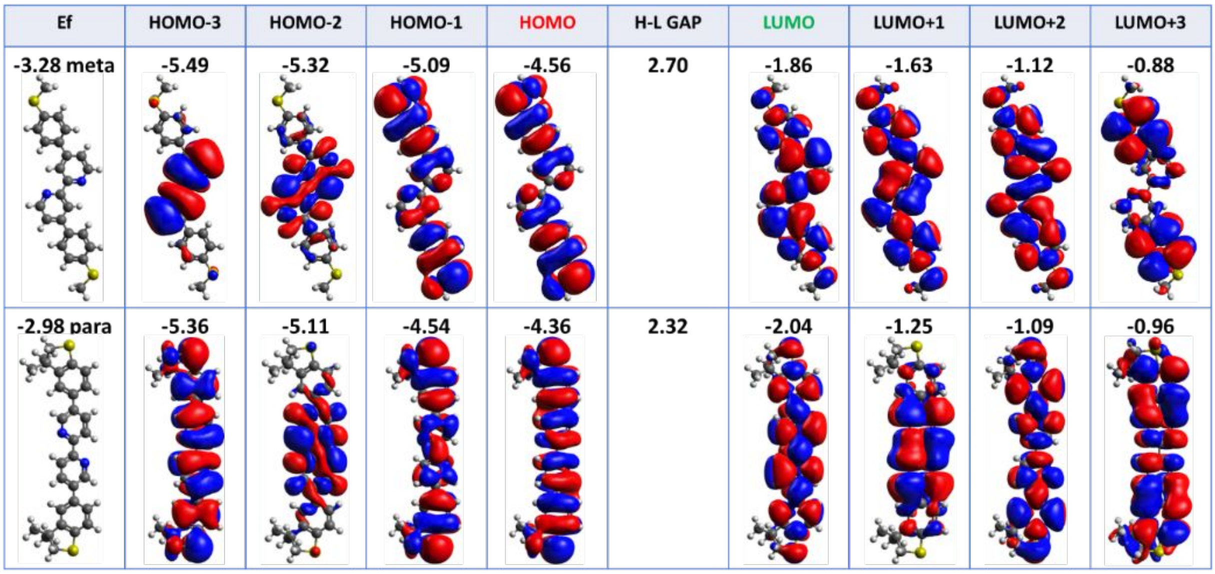

Figure S22. DFT calculated orbital orbitals with corresponding energy values of L^{meta} and L^{para} molecules. Isocontours at 0.055 e bohr$^{-3/2}$

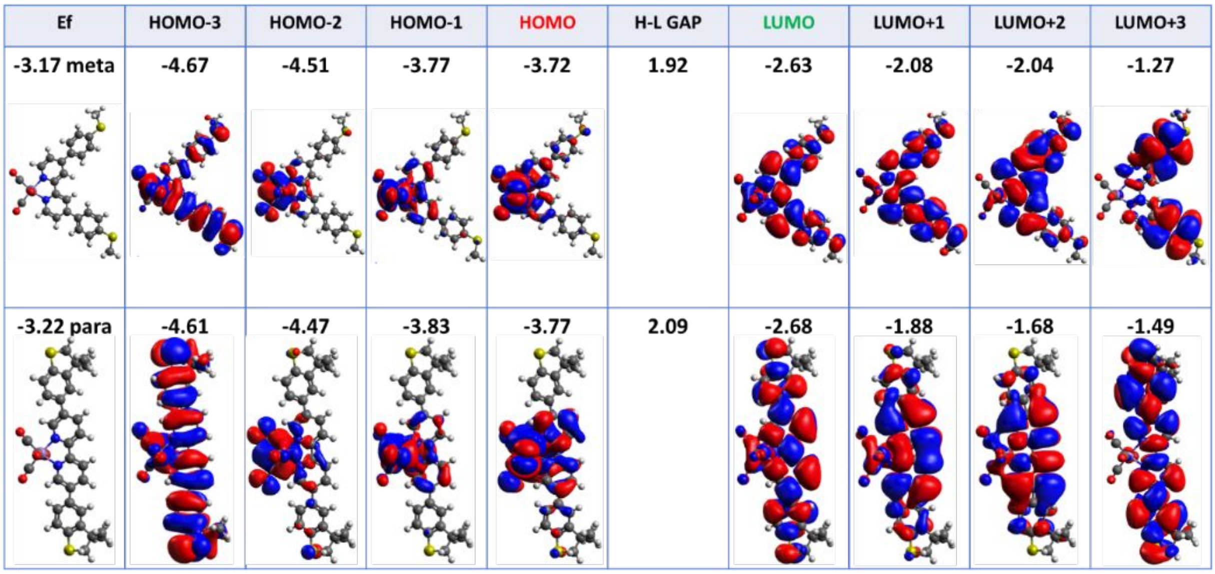

Figure S23. DFT calculated orbitals with corresponding energy values of Mn^{meta} and Mn^{para} molecules. Isocontours at 0.055 e bohr$^{-3/2}$

Ef	HOMO-3	HOMO-2	HOMO-1	HOMO	H-L GAP	LUMO	LUMO+1	LUMO+2	LUMO+3
-3.19 para	-4.63	-4.10	-4.02	-3.74	1.11	-2.63	-1.85	-1.63	-1.41

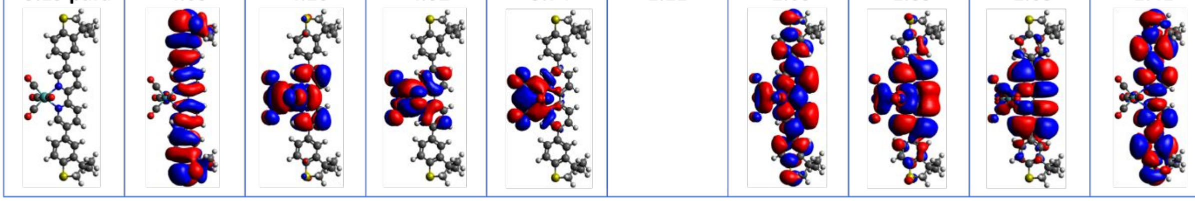

Figure S24. DFT calculated orbitals with corresponding energy values of the Mo^{para} molecule. Isocontours at 0.055 e $bohr^{-3/2}$

Electrical Conductance

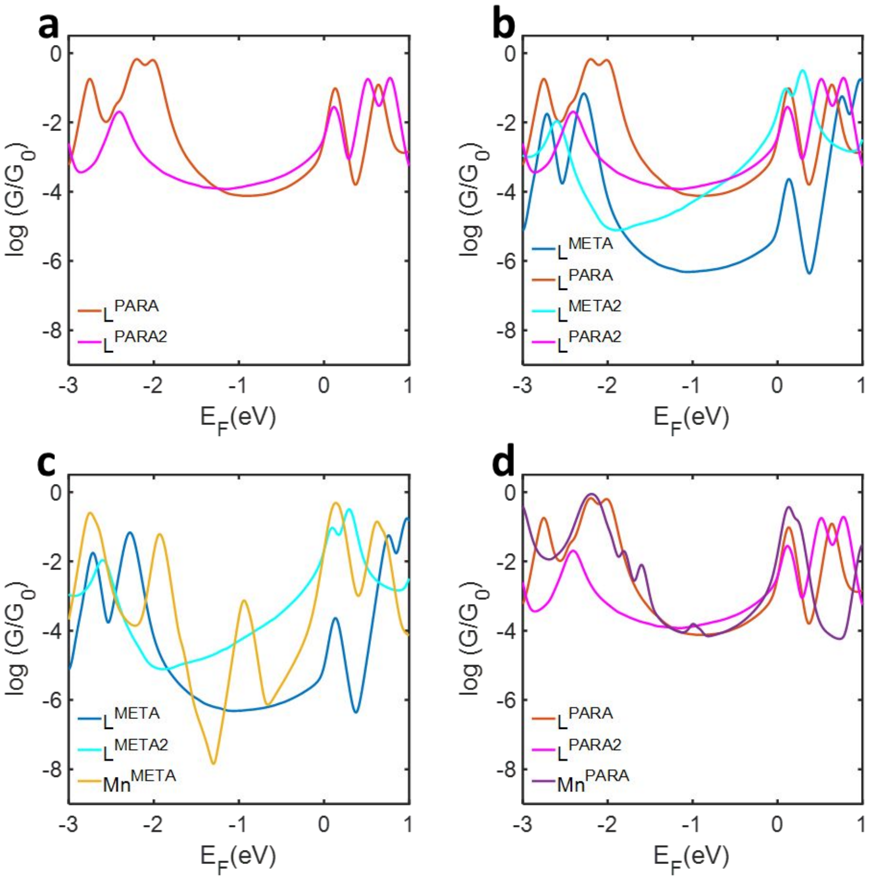

Figure S25. Electrical conductance curve (a) L^{para} and $L^{para}2$ (b) L^{meta}, L^{para}, $L^{meta}2$ and $L^{para}2$ (c) L^{meta}, $L^{meta}2$ and Mn^{meta} (d) L^{para}, $L^{para}2$ and Mn^{para}.

Transmission Curves

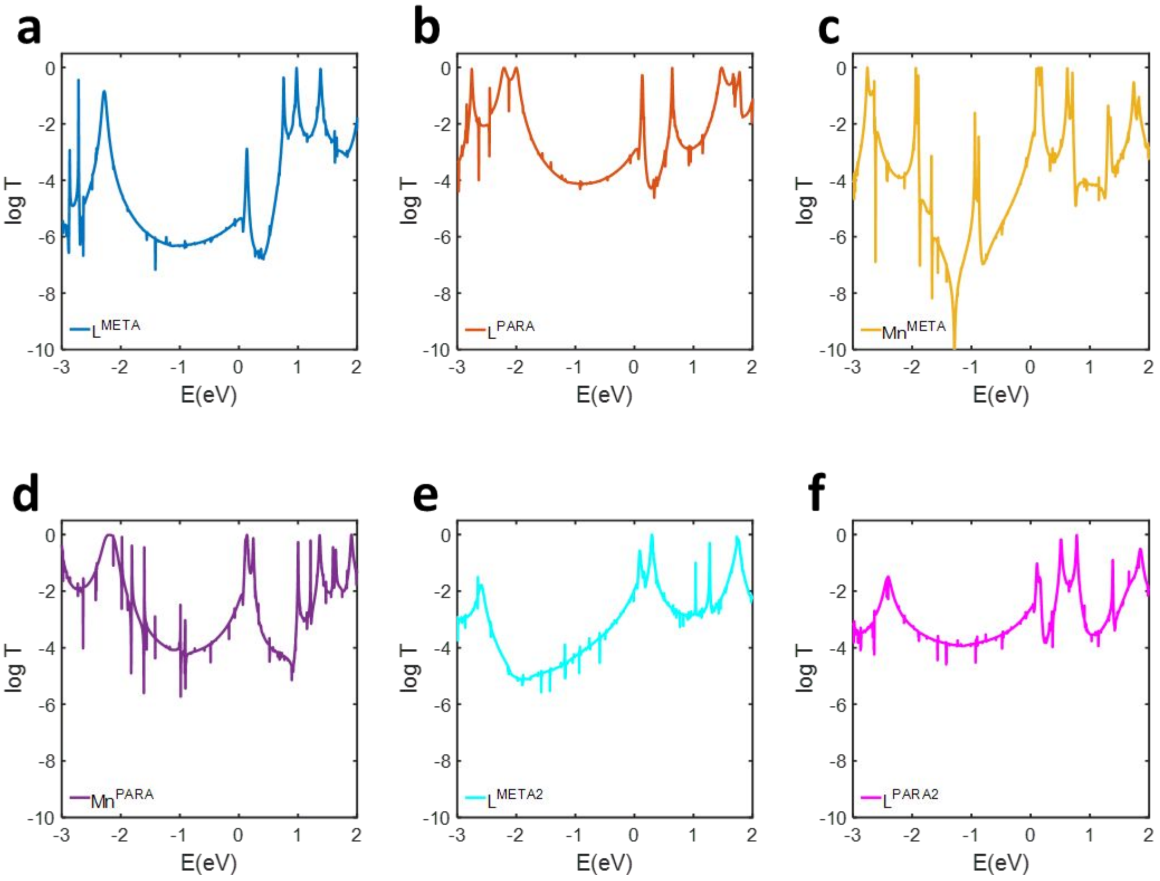

Figure S26. Transmission coefficients for (a) L^{meta}, (b) L^{para}, (c) Mn^{meta} (d) Mn^{para} (e) $L^{meta}2$, and (f) $L^{para}2$.

Proposed shorter molecules

To compensate for the low conductance of the compounds measured in this paper we propose an analogous series of compounds (see Figure S27) where the anchor groups are directly attached to the pyridyl rings. Shown in Figure S28 are their theoretical conductance values, each showing increased conductance to their longer analogues. This is an important consideration for the development of related systems.

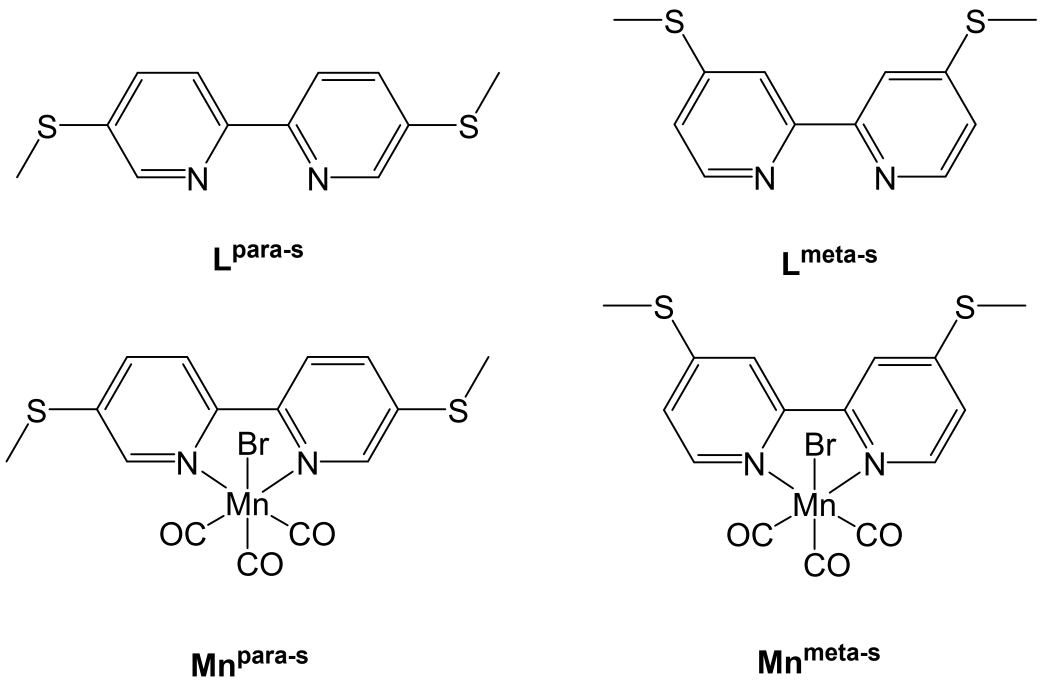

Figure S27. Proposed shorter analogous compounds.

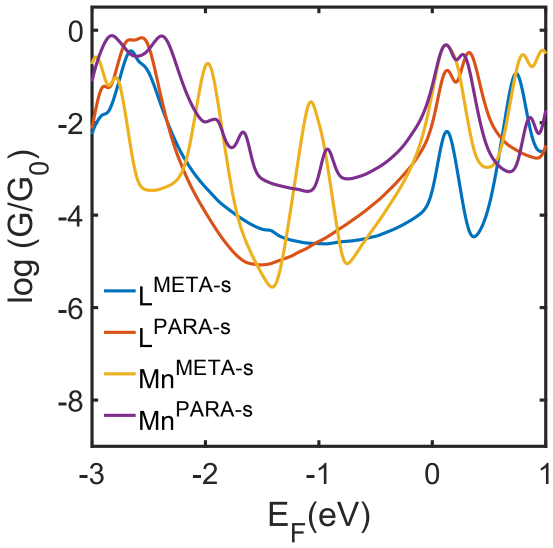

Figure S28. DFT calculated room-temperature electrical conductance for the shortened molecules L^{meta-s}, L^{para-s}, Mn^{meta-s} and Mn^{para-s}.

Computational Methods

DFT Calculation – The geometry of each structure studied in this paper was relaxed to the force tolerance of 10 meV/Å using the SIESTA[6] implementation of DFT, with a double-ζ polarized basis set (DZP) and the Generalized Gradient Approximation (GGA) functional with Perdew–Burke–Ernzerhof (PBE) parametrization. A real-space grid was defined with an equivalent energy cutoff of 250 Ry. We then calculate spin polarized molecular orbitals and spin density of gas phase molecules.

Spin Transport – To calculate the electronic properties of the device, from the converged DFT calculation, the underlying spin polarized mean-field Hamiltonian Hσ was obtained where σ = ↑, ↓ and ↑ (↓) denotes majority (minority) spin. Hσ was combined with our quantum transport code, GOLLUM[7]. This yields the spin-dependent transmission coefficient Tσ(E) for electrons of energy E (passing from the source to the drain) via the relation Tσ(E) = $Tr(\Gamma\sigma \mathrm{L}(E) G \mathrm{R}\sigma(E) \Gamma\sigma \mathrm{R}(E) G \mathrm{R}\dagger\sigma(E))$ where $\Gamma\sigma \mathrm{L,R}(E) = i(\sum\sigma \mathrm{L,R}(E) - \sum\sigma \mathrm{L,R}\dagger(E))$ describes the level broadening due to the coupling between left L and right R electrodes and the central scattering region, ΣL,Rσ(E) are the retarded self-energies associated with this coupling and $G\mathrm{R}\sigma = (ES - H - \sum\sigma \mathrm{L} - \sum\sigma \mathrm{R}) - 1$ is the retarded Green's function, where Hσ is the Hamiltonian and S is the overlap matrix obtained from SIESTA implementation of DFT. The total transmission is then calculated from T(E) = (T↑ + T↓)/2.

Electrical Conductance – Using the approach explained in ref[8], the electrical conductance is calculated from Landaure's formula $G = G0\int + \infty - \infty dET(E)(-\partial f(E,T,E\mathrm{F})/\partial E)$, where $f = (e(E - E\mathrm{F})/k\mathrm{B}T + 1) - 1$ f is the Fermi–Dirac probability distribution function, T is the temperature, EF is the Fermi energy, G0 = 2e2/h is the conductance quantum, e is the electron charge, and h is the Planck's constant.

S4. References

1. Meisner, J. S.; Sedbrook, D. F.; Krikorian, M.; Chen, J.; Sattler, A.; Carnes, M. E.; Murray, C. B.; Steigerwald, M.; Nuckolls, C. Functionalizing molecular wires: a tunable class of α,ω-diphenyl-μ,ν-dicyano-oligoenes. *Chemical Science* **2012,** 3 (4), 1007-1014 DOI: 10.1039/C2SC00770C.

2. Neumann, S.; Wenger, O. S. Fundamentally Different Distance Dependences of Electron-Transfer Rates for Low and High Driving Forces. *Inorganic Chemistry* **2019,** 58 (1), 855-860 DOI: 10.1021/acs.inorgchem.8b02973.

3. Dolomanov, O. V.; Bourhis, L. J.; Gildea, R. J.; Howard, J. A. K.; Puschmann, H. OLEX2: a complete structure solution, refinement and analysis program. *Journal of Applied Crystallography* **2009,** 42, 339-341 DOI: 10.1107/s0021889808042726.

4. Sheldrick, G. M. *SHELXL: Suite of Programs for Crystal Structure Analysis*, Tammanstrasse 4: Gottingen, 1998.

5. M. J. Frisch; G. W. Trucks; H. B. Schlegel; G. E. Scuseria; M. A. Robb; J. R. Cheeseman; G. Scalmani; V. Barone; B. Mennucci; G. A. Petersson; H. Nakatsuji; M. Caricato; X. Li; H. P. Hratchian; A. F. Izmaylov; J. Bloino; G. Zheng; J. L. Sonnenberg; M. Hada; M. Ehara; K. Toyota; R. Fukuda; J. Hasegawa; M. Ishida; T. Nakajima; Y. Honda; O. Kitao; H. Nakai; T. Vreven; J. A. Montgomery, J., J. E. Peralta, F. Ogliaro, M. Bearpark, J. J. Heyd, E. Brothers, K. N. Kudin, V. N. Staroverov, R. Kobayashi, J. Normand, K. Raghavachari, A. Rendell, J. C. Burant, S. S. Iyengar, J. Tomasi, M. Cossi, N. Rega, J. M. Millam, M. Klene, J. E. Knox, J. B. Cross, V. Bakken, C. Adamo, J. Jaramillo, R. Gomperts, R. E. Stratmann, O. Yazyev, A. J. Austin, R. Cammi, C. Pomelli, J. W. Ochterski, R. L. Martin, K. Morokuma, V. G. Zakrzewski, G. A. Voth, P. Salvador, J. J. Dannenberg, S. Dapprich, A. D. Daniels, Ö. Farkas, J. B. Foresman, J. V. Ortiz, J. Cioslowski, and D. J. Fox. *Gaussian 09*, A.1; Gaussian, Inc: Wallingford CT, 2009.

6. Soler, J. M.; Artacho, E.; Gale, J. D.; Garcia, A.; Junquera, J.; Ordejon, P.; Sanchez-Portal, D. The SIESTA method for ab initio order-N materials simulation. *Journal of Physics: Condensed Matter* **2002,** 14 (11), 2745-2779 DOI: 10.1088/0953-8984/14/11/302.

7. Ferrer, J.; Lambert, C. J.; Garcia-Suarez, V. M.; Manrique, D. Z.; Visontai, D.; Oroszlany, L.; Rodriguez-Ferradas, R.; Grace, I.; Bailey, S. W. D.; Gillemot, K.; Sadeghi, H.; Algharagholy, L. A. GOLLUM: a next-generation simulation tool for electron, thermal and spin transport. *New Journal of Physics* **2014,** 16, DOI: 10.1088/1367-2630/16/9/093029.

8. Sadeghi, H. Theory of electron, phonon and spin transport in nanoscale quantum devices. *Nanotechnology* **2018,** 29 (37), 373001 DOI: 10.1088/1361-6528/aace21.
